# Metabolic synergy in Camelina reproductive tissues for seed development

**DOI:** 10.1126/sciadv.abo7683

**Published:** 2022-10-28

**Authors:** Somnath Koley, Kevin L. Chu, Thiya Mukherjee, Stewart A. Morley, Anastasiya Klebanovych, Kirk J. Czymmek, Doug K. Allen

**Affiliations:** ^1^ Donald Danforth Plant Science Center, St. Louis, MO, USA.; ^2^ United States Department of Agriculture-Agricultural Research Service, Donald Danforth Plant Science Center, St. Louis, MO, USA.

## Abstract

Photosynthesis in fruits is well documented, but its contribution to seed development and yield remains largely unquantified. In oilseeds, the pods are green and elevated with direct access to sunlight. With ^13^C labeling in planta and through an intact pod labeling system, a unique multi-tissue comprehensive flux model mechanistically described how pods assimilate up to one-half (33 to 45%) of seed carbon by proximal photosynthesis in *Camelina sativa*. By capturing integrated tissue metabolism, the studies reveal the contribution of plant architecture beyond leaves, to enable seed filling and maximize the number of viable seeds. The latent capacity of the pod wall in the absence of leaves contributes approximately 79% of seed biomass, supporting greater seed sink capacity and higher theoretical yields that suggest an opportunity for crop productivity gains.

## Teaser

Flux analysis indicates that camelina pod photosynthesis contributes to seed yield.

## INTRODUCTION

**Fig. 1.**
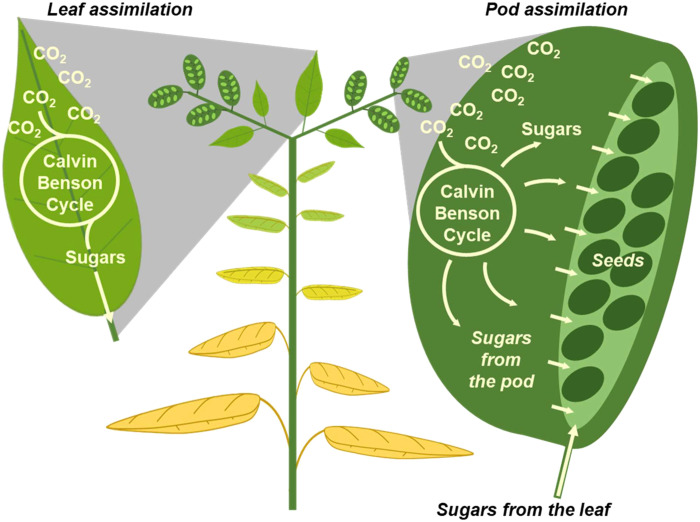
Illustration highlighting potential routes to supply nutrients for oilseed metabolism. Green leaves and pod walls function as source tissues for filling seeds.

In a field of oilseeds (e.g., canola or camelina), a sea of bold yellow flowers gives way to strong green hues from the pods (fruits) in the summer, with most green leaves being just a distant memory having long since shriveled and disappeared. This observation suggests that the pods themselves might provide photosynthate at the crucial late-developmental stage when seeds are filling (Fig. 1), a theory that is supported by studies in pod-bearing plants (*1*–*5*) investigated by shading (*3*, *6*), transcriptome (*7*), and proteome evaluations (*8*) and described through fruit metabolite levels with high-resolution imaging (*9*). Broadly, investigations of other species (*10*–*14*) have hypothesized roles for plant reproductive architecture; however, analyses of the contribution of pod photosynthesis to grain yield have not included comprehensive studies of metabolic flux. More generally, flux analyses describing the synergy of multiple tissues have not been reported for any biological system; thus, unanswered questions remain regarding the agricultural relevance of pod photosynthetic metabolism to final seed yield.

Oil and protein are important storage reserves in seeds, although producing these reduced forms of carbon is energetically costly. Most stored lipids are triacylglycerols, composed of fatty acids that are generated by combining 2-carbon acetyl groups derived from 3-carbon pyruvate. When pyruvate is decarboxylated to acetyl coenzyme A, 33% of the carbon is converted to CO_2_. Seeds also respire carbon through the tricarboxylic acid (TCA) cycle to produce adenosine triphosphate and carbon skeletons for amino acids used in protein biosynthesis. As a result, CO_2_ concentrations within the seed can be 600- to 2000-fold higher than the ambient level (*15*). Some seeds use Ribulose bisphosphate carboxylase-oxygenase (Rubisco) to reassimilate metabolic CO_2_, improving carbon efficiency (*16*–*19*), although others do not (*20*, *21*), leaving open questions about the contribution of other reproductive tissues including pod walls that are green and encase the seeds. Given that seeds provide the agronomic value of most crops, any contribution to seed biomass from coordinated metabolism within reproductive tissues would have implications for strategies to increase grain yield.

In this study, the substantial contribution of pod wall photosynthesis to seed biomass and the untapped potential for higher crop productivity were demonstrated and quantified in *Camelina sativa*, an emerging oilseed crop. Camelina seeds were evaluated at mid-development when oil and protein production are substantial. Fluorescent dye confirmed that metabolites could move from the pod wall surface to the developing seed. The network of metabolic interactions between reproductive components was detailed with isotopic tracers in planta and through a unique intact pod labeling system and computational multi-tissue flux model that quantitatively describes metabolic coordination in seed filling.

## RESULTS AND DISCUSSION

### Camelina pod walls are photosynthetically active

**Fig. 2.**
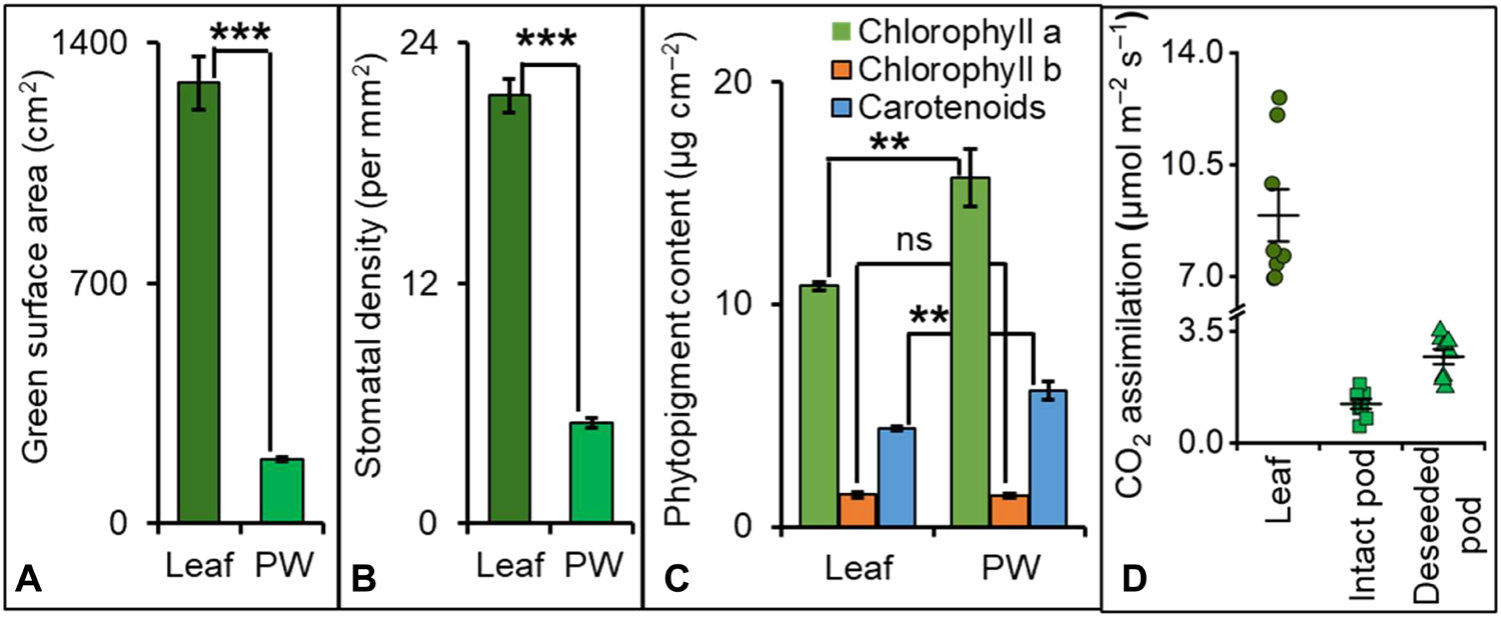
Comparison of photosynthetic variables between leaves and pod walls. Leaves and pod walls (PW) were assayed at 15 days after flowering (DAF) for (**A**) green surface area, (**B**) number of stomata per unit area, and (**C**) amount of chlorophyll a, b, and total carotenoids. For calculation of leaf green surface area and phytopigment content, LM to LR leaves (fig. S1) were included but senescing leaves were excluded (results presented in data S1). (**D**) Net CO_2_ assimilation of camelina plant tissues at 400 μmol m^−2^ s^−1^ light intensity experienced in the greenhouse. The deseeded pod wall denotes an intact pod wall without any seeds. The results are means ± SE [*n* = 3 for (A) and (C); *n* = 8 for (B) and (D)]. Student’s *t* tests were used for statistical analyses between leaves and pod walls; **P < 0.005, ***P < 0.001, and ns indicates no statistical differences at *P* = 0.05.

During seed filling, leaves at the bottom half of camelina plants were yellow in color, indicating a reduced photosynthetic capacity, although reproductive structures including the pod wall remained dark green (fig. S1). The pods were at the top of the plant where access to sunlight is unimpeded by the canopy. The surface area and stomatal density of pod walls were 15 and 24% of green leaf values, respectively, but chlorophyll and carotenoid levels were elevated in pod walls (Fig. 2, A to C, fig. S2, and data S1), indicating that the smaller surface area of reproductive structures could still efficiently use light for seed development. We sought to quantify the capacity of pod walls to assimilate CO_2_. Intact pod assimilation measurements may significantly underestimate carboxylation rates of outer tissues because of CO_2_ evolution within the pod structure (*22*); therefore, photosynthesis by the pod wall was analyzed in the presence or absence of seeds (i.e., intact versus deseeded pods). When light was exclusively supplied to deseeded pods, the net assimilation was between 14 and 51% (average 30%) of green leaves and twice that of an intact pod per unit surface area (Fig. 2D), providing an initial estimate of the net photosynthesis of the pod wall that includes the CO_2_ exhaust flux from developing seeds.

### Development of an experimental approach to assess carbon exchange between pod components

**Fig. 3.**
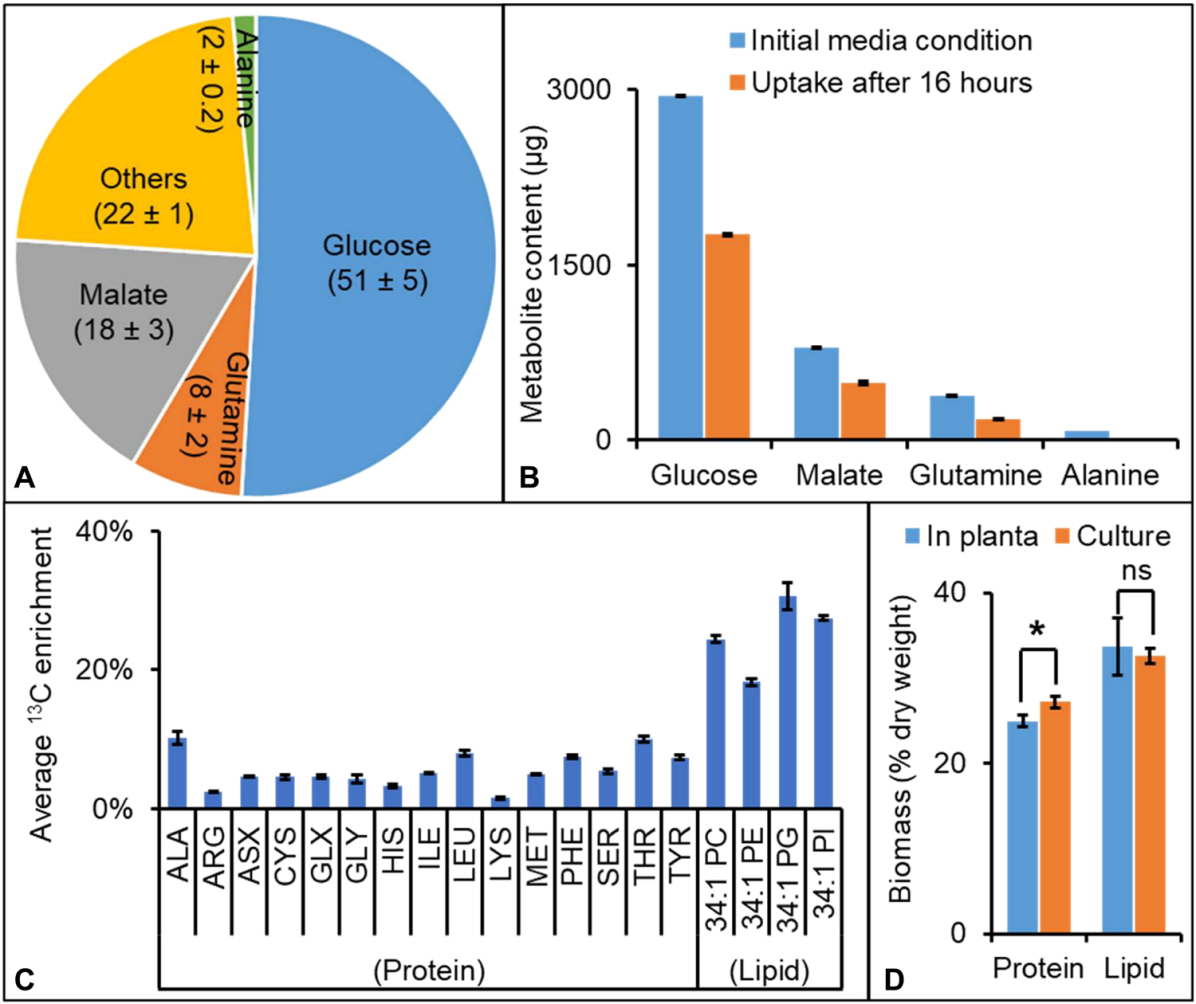
Pod culturing approach to assess carbon exchange between organs. All results are means ± SE [*n* = 6 for (A) and (B); *n* = 3 for (C) and (D)]. All abbreviations are listed in data S3. (**A**) Culturing conditions were based on measurement of metabolite levels (molar percentage) in phloem sap from a seed-bearing branch at the 15 DAF stage (full metabolite list measured from phloem sap presented in data S2). (**B**) Uptake of medium substrates after 16 hours of culturing. (**C**) Assessment of biomass production after 32 hours of culturing pods with U-^13^C glucose and measurement of isotope incorporation in proteinogenic amino acids and lipids that comprise cotyledonary biomass storage reserves. (**D**) Seed biomass comparison between in planta and 6-day cultured pods. Student’s *t* tests were used for statistical analyses of biomass content between in planta and culture; *P < 0.05 and ns means statistically insignificant at *P* = 0.05.

Isotopic tracers can assess pathway activity and flux and establish the partitioning of nutrients that enables cellular metabolism (*23*–*26*). In oilseeds, isotopes are frequently used to characterize embryo storage metabolism (*17*, *24*, *27*–*29*); however, seed culturing techniques fail to capture coordinated metabolism with other reproductive tissues. Intact pod culturing techniques are limited in number (*5*, *9*, *30*) and thus emergent properties resulting from shared tissue metabolism remain unresolved. To specifically examine the metabolic synergy between tissues, a pod labeling system was developed including nutrient supply that matched in planta conditions and seed biomass production (fig. S3). Glucose, malate, glutamine, and alanine were major constituents of the phloem sap of seed-bearing branches (Fig. 3A and data S2) and therefore were used as carbon and nitrogen sources in the pod culturing system.

The efficacy of the in pod culturing system was assessed using different strategies. At 16 hours, 30 to 60% of the carbon and nitrogen substrates had been taken up by the pod (Fig. 3B); therefore, the medium was replaced at 16 hours for longer culturing periods. Biomass production in cultured pods was monitored by tracing isotope incorporation in cotyledonary protein and lipid fractions from U-^13^C glucose provision. By 32 hours, proteinogenic amino acids and phospholipids had notable labeling, indicating that the cotyledon continued to grow and assimilate reserves during the culturing period (Fig. 3C). For example, phosphatidylcholine (PC), the first labeled lipid from ^14^C acetate provision to plant cells and a non-Kennedy pathway intermediate for TAG production and membrane lipids (*25*, *31*, *32*), reached 24% average ^13^C labeling. A similar growth rate was observed with in planta and cultured pods as verified by comparison of total seed protein and lipid levels following an extended culturing period of 6 days (Fig. 3D).

### Metabolites from the phloem and pod wall–assimilated CO_2_ contribute to pod metabolism

**Fig. 4.**
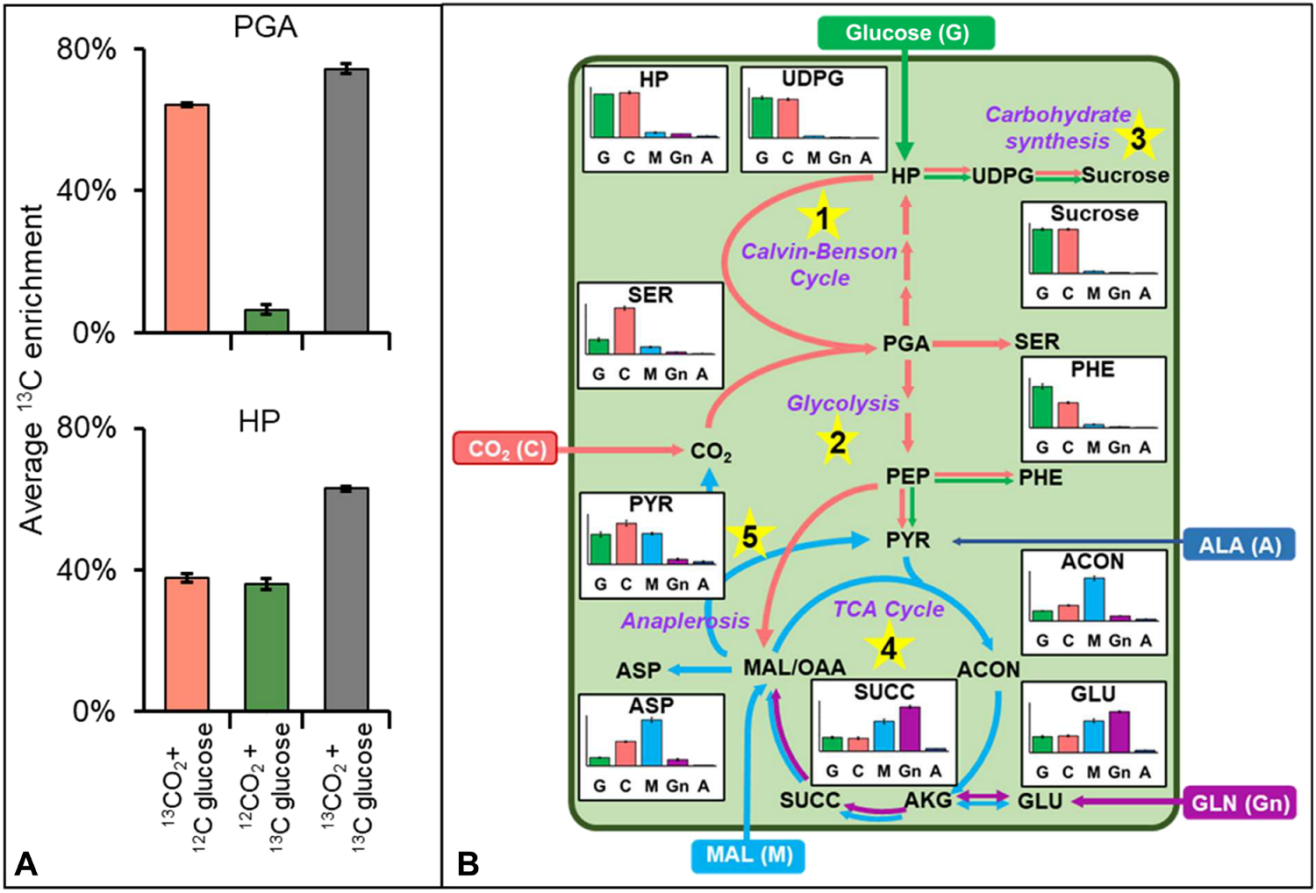
Detailed analysis of pod wall metabolism using different ^13^C sources. (**A**) ^13^C enrichment in PGA and hexose phosphate (HP) after 4 hours of culturing from independent or combined sources of ^13^CO_2_ and U-^13^C glucose. The results are means ± SE (*n* = 2) where four biological replicates were pooled to precisely measure low abundant metabolites. (**B**) Relative ^13^C enrichment (*y* axis) in pod wall metabolites after 16 hours of culturing from ^13^C sources including CO_2_, glucose, malate, glutamine, or alanine. Five different pathways are described: (1) Calvin Benson Cycle (CBC) represented by serine (SER) that is a close product of PGA; (2) a combination of glycolysis and CBC represented by HP and phenylalanine (PHE); (3) carbohydrate biosynthesis represented by UDP-glucose (UDPG) and sucrose; (4) TCA cycle represented by *cis*-aconitate (ACON), glutamate (GLU), succinate (SUCC), and aspartate (ASP); and (5) anaplerosis represented by pyruvate (PYR) labeling. Assorted colors represent the five sources, with the arrow color indicating the primary source used in a particular pathway. All abbreviations used are listed in data S3. The results are means ± SE (*n* = 3).

Photosynthesis by the pod wall was tracked by supplying ^13^CO_2_ to intact pods under light or dark conditions and tracing label incorporation through quantification using liquid chromatography tandem mass spectrometry (LC-MS/MS). The measurements were compared with provision of U-^13^C glucose through the petiole and unlabeled CO_2_ or the combined provision of ^13^CO_2_ and U-^13^C glucose. Phosphoglyceric acid (PGA), the immediate photosynthetic product from Rubisco, was significantly labeled from the ^13^CO_2_ study in the light (64%; Fig. 4A) while little ^13^C-PGA (<4.1%) was detected in the dark (data S4) or from ^13^C-glucose provision (~6%; Fig. 4A). The combined supply of organic and inorganic isotopic carbon resulted in 74% enrichment in PGA that was comparable to the summed value from labeling with individual sources, indicating that photosynthetic assimilation in the pod wall is substantial. Hexose phosphates (e.g., fructose 6-phosphate and glucose-6-phosphate) are derived from the Calvin Benson Cycle (CBC) in the chloroplast (which can then be exported to the cytosol through triose phosphate exchange) or are generated in the cytosol from the conversion of the imported sugars through the phloem. Labeling in hexose phosphates exhibited comparable enrichment (36 to 38% average ^13^C) from both ^13^CO_2_ and ^13^C-glucose and was nearly doubled with supply of combined ^13^C sources, indicating that assimilated CO_2_ accounted for a similar amount of carbon as sugars from the phloem.

To elucidate the contribution of all phloem-supplied carbon sources, glucose, malate, glutamine, and alanine were individually substituted with a ^13^C-enriched form and contrasted with labeling from CO_2_ (Fig. 4B and fig. S4). Whereas substantial enrichment from ^13^CO_2_ provision was detected in intermediates related to carbon assimilation (e.g., serine that is metabolically close to PGA, product of Rubisco), other detected labeled intermediates including hexose phosphates, nucleotide sugars [uridine diphosphate glucose (UDPG)], sucrose, and amino acids (e.g., phenylalanine) were products of both glucose and CO_2_ sources. Organic acid production was characterized by labeling of intermediates mostly from labeled malate and glutamine substrates. Pyruvate, a vital connecting node in central metabolism, became labeled with the provision of ^13^C-malate, indicating anaplerosis involving malic enzyme. The combination of experiments suggested that the pod wall used disparate carbon sources including organic carbon from the phloem sap and inorganic carbon from the atmosphere.

### Photosynthetic pod walls provide local delivery of carbon to developing seeds

Major components of a seed include the seed coat, endosperm, and cotyledons. By maturity, most of the biomass is contained within the cotyledons in oilseed crops. Isotope analyses were extended to developing seeds to evaluate carbon import from the phloem and pod wall to seed tissues. ^13^C enrichment in cotyledons from the supply of U-^13^C–based phloem metabolites (i.e., glucose, malate, glutamine, and alanine) provided the contribution from vegetative tissues. The influence of pod wall photosynthesis on seed filling was assessed with the provision of ^13^CO_2_ to intact pods or partially opened pods that were severed to allow direct ^13^CO_2_ access to the pod cavity containing the seeds (fig. S5). There were no significant labeling differences in the seed metabolites from the two conditions. Furthermore, seed respiration was similar in the absence or presence of physiological light levels transmitted to the seed [~20 μmol m^−2^ s^−1^; (*17*, *21*)]. The combined results are consistent with seed metabolism that is mostly heterotrophic (data S5) and with the absence of stomata on seed surface (fig. S2D). Thus, ^13^C incorporation in cotyledons from an external ^13^CO_2_ supply to the intact pods was the result of photosynthetic pod wall–based assimilation and not substantial photosynthesis by the seed itself.

**Fig. 5.**
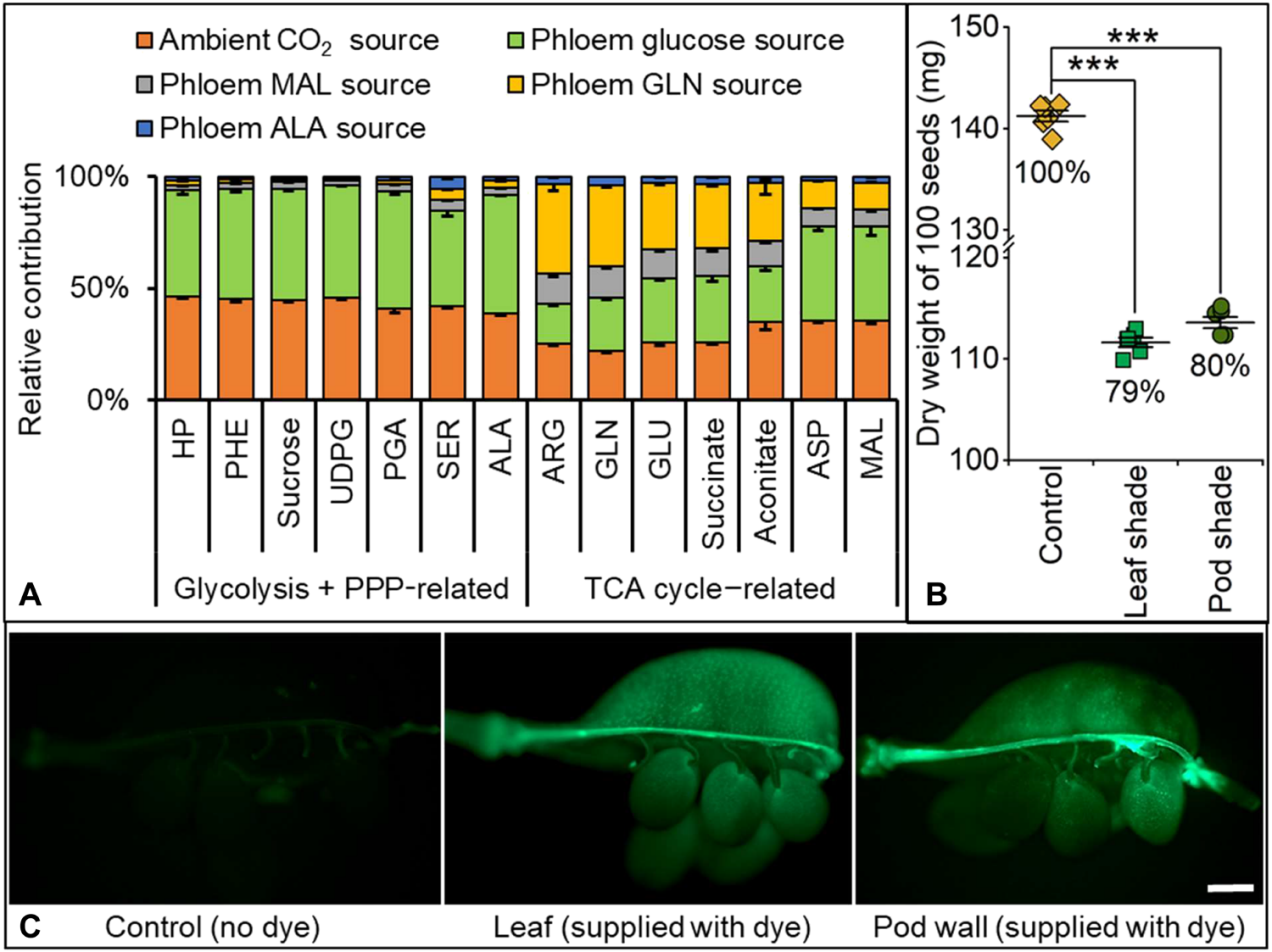
Participation of pod wall and phloem sap in seed metabolism. (**A**) Relative contribution of ambient CO_2_ and four exogenous metabolites from phloem sap was assessed from ^13^C enrichment in cotyledon central carbon metabolites after 16 hours of culturing with independent supply of ^13^C sources, i.e., ^13^CO_2_, U-^13^C glucose, malate, glutamine, and alanine (means ± SE, *n* = 3). (**B**) Compared to the control (nonshaded plants), relative seed yield decreased after shading all leaves or pods at the beginning of seed development (means ± SE, *n* = 3). Three biological replicates contained 100 seeds each. Student’s *t* tests were used for statistical analyses of seed yield between control and shaded leaves or pods; ***P < 0.001 while differences in seed yield are statistically insignificant between the two shading studies. (**C**) Microscopy detection of fluorescent dye in pod wall and seeds after 10 hours of Lucifer yellow loading onto the upper surface of the leaf or exterior surface of the pod wall. No dye was provided to the control pod wall sample. Scale bar, 1000 μm.

Isotope tracers supplied individually to intact pods entered the metabolic network of cotyledons at different nodes, contributing to distinct aspects of seed metabolism (Fig. 5A). Phosphorylated intermediates associated with sugar metabolism, glycolysis, and pentose phosphate pathway were heavily labeled from ^13^CO_2_ and ^13^C-glucose in cotyledons. Other carbon sources resulted in notable labeling of organic acids and primary intermediates associated with TCA metabolism. In total, pod wall photoassimilates contributed 22 to 46% of the carbon received by the seed with the remaining supply coming from vegetative tissues. Camelina pod wall growth had ceased by the time seed filling ensues (data S6) and thus pod wall photoassimilates are dedicated for seed metabolism. In other Brassicaceae, production of storage reserves in seeds is broadly correlated with photosynthetic activity of the outer silique wall but not with leaves (*33*, *34*), and pods grown under elevated light conditions tend to have increased oil content (*35*).

To validate the substantial contribution of the photosynthetic pod wall to seed metabolism, camelina leaves or pods present at the beginning of seed development were shaded (fig. S6). After 45 days, seed biomass was reduced but comparable for both shade treatments (79 to 80% of unshaded plants; Fig. 5B), indicating that both pod walls and leaves contribute significantly to seed biomass and suggesting untapped potential for higher plant productivity. Although leaves are major providers of carbon during early reproductive organ development (*36*), the onset of senescence at midseed maturation compromises photoassimilate delivery from leaves at the time when seeds are filling. The developmental timing of pod formation is inherently linked to seed filling unlike leaves that are continuously produced to support roots, stems, and other sinks. The development of pods on an inflorescence is a continuous process during the second half of the plant life cycle due to the indeterminate flowering of camelina; thus, the role of pod walls in producing viable seeds is likely to be further increased in later-emerging pods when photosynthetic leaf area is severely diminished because of senescence.

To evaluate the vascular flow of metabolites from pod wall to seed, Lucifer yellow dye was applied to abraded pod surfaces and its movement was tracked over time (fig. S7A), with dye application to a leaf surface serving as a control. The dye was detectable in the inner seed tissues within 10 hours, confirming a route for metabolite movement from the pod wall to the seed (Fig. 5C and fig. S7B). Dye also moved from the leaf to the pod wall and seed in the plants with dye-treated leaves. Although the dye moved from the pod wall to attached seeds and the internode between pod and pedicel, the dye was not detected in adjacent pods (fig. S7), indicating that flux from the phloem is unidirectional and precluded flow of metabolites between pods. The results further confirmed pod function as a local source of nutrients for enclosed seeds.

### Sucrose and glutamine are the primary metabolites transported from pod walls to developing seeds

**Fig. 6.**
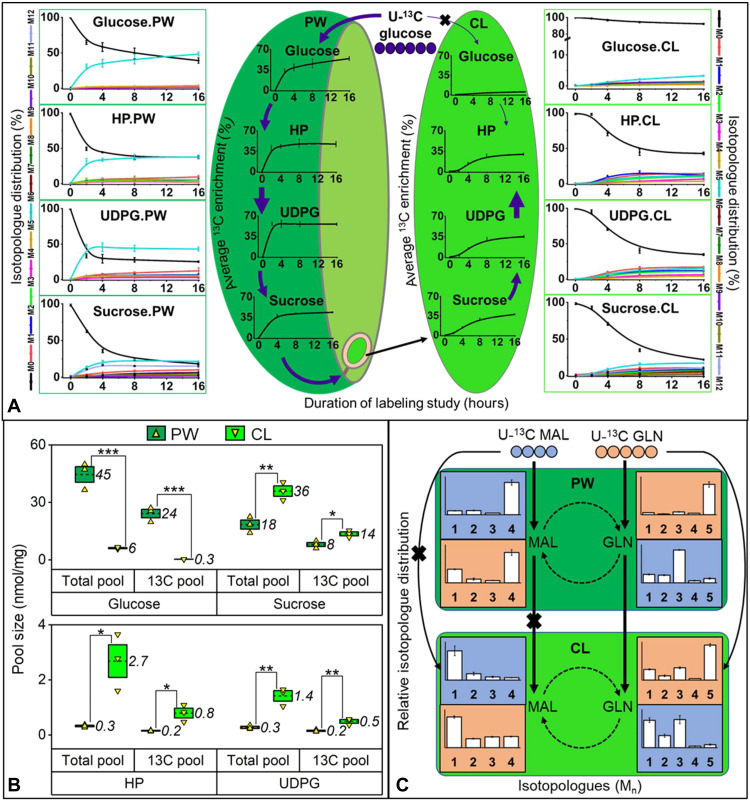
Determination of metabolite transport from the pod wall to cotyledon. (**A**) Transport of sucrose from the analysis of trends in ^13^C enrichment and isotopologue distributions of glucose, HP, UDPG, and sucrose from PW and cotyledon (CL) over 16 hours of U-^13^C glucose supply. (**B**) Validation of sucrose transport from total and ^13^C pool size after 16 hours of labeling with U-^13^C glucose. Boxes represent SEM. Student’s *t* tests were used for statistical analyses of pool sizes between PW and CL; *P < 0.05, **P < 0.005, and ***P < 0.001. (**C**) Transport of glutamine (GLN), not malate (MAL), established by trends in isotopologue distributions (M0 not shown) of MAL and GLN in pod tissues after 16 hours of labeling from an independent supply of U-^13^C MAL and GLN. The results are presented as means ± SE (*n* = 3).

The export of sugars, organic acids, and amino acids from the pod wall to the cotyledon was tracked over time by comparing labeling in central intermediates from each tissue. Pod wall glucose, hexose phosphates, UDPG, and sucrose from the U-^13^C glucose experiment became significantly labeled within 2 hours, achieved isotopic steady state by 4 hours, and did not change significantly through the remainder of the 16-hour labeling period (Fig. 6A). The fully labeled isotopologue (M6 of C6 compounds or M6/M12 of C12 compounds) was the highest in proportion to other isotopologues. Thus, phloem-derived sugar was used for the direct assembly of phosphorylated intermediates in the pod wall and resulted in sucrose production. Similar ^13^C enrichment by 16 hours in sucrose pools from the pod wall and cotyledons revealed the direct transport of this sugar from the pod wall to the cotyledon. The initial lag in sucrose labeling in cotyledons indicated that the pod wall was the precursor supply of sugar that was transported between two spatially distinct compartments through an endosperm sucrose transfer pool. The characteristic lag was repeated in phosphorylated intermediates of the cotyledon, consistent with their biosynthesis from the imported sucrose.

In contrast, there was little isotopic incorporation into cotyledonary glucose (5% ^13^C after 16 hours of labeling), indicating little direct transport from phloem to the cotyledons. Unlike the pod wall, M6 of hexose phosphates and UDPG was not the highest isotopologue observed in cotyledons, signifying that labeled metabolites in cotyledons were not generated directly from phloem glucose that was fully labeled, but the result of interconversion due to pod wall photosynthesis. Reduced labeling in metabolites can sometimes be confounded with dilution because of large pool sizes that do not turn over quickly; therefore, metabolite pool sizes in the individual tissues were compared (Fig. 6B). Relative to the pod wall, glucose was present at low levels in the cotyledon. High sucrose-hexose ratios have been reported in seeds during storage product accumulation (*37*, *38*). In cotyledons, the average isotopic enrichment of glucose was one-seventh that of sucrose (Fig. 6A) although the glucose pool size was also comparatively smaller (~6-fold reduction; Fig. 6B). Thus, the calculated absolute ^13^C sucrose pool was 43-fold greater than glucose in cotyledons, validating that sucrose is the major seed-imported sugar that is transported from the pod wall. Pool sizes of sucrose, hexose phosphates, and UDPG were larger in cotyledons, consistent with a sink metabolic role that has a high carbon demand (Fig. 6B).

The movement of sucrose between pod tissues was further supported by ^13^C-sucrose labeling studies supplied through the pedicel for 16 hours. If this sugar was directly moved from the phloem to inner seed tissues, M12 would be expected to be the lone isotopologue. In the labeling study, M6 and M12 isotopologues were elevated in all three measured pod components (pod wall, endosperm, and cotyledon; fig. S8A); however, other isotopologues were also detectable as a result of bond rearrangements involving carbon assimilated in the pod wall from an unlabeled CO_2_ source. In addition, the similar labeling distributions between pod wall, liquid endosperm, and cotyledons indicated that sucrose was transferred from the pod wall to cotyledon via endosperm, but phloem sugar was not directly transported to the camelina seed tissues. Labeling experiments with ^13^CO_2_, U-^13^C_4_-malate, or U-^13^C_5_-glutamine confirmed the match in pod wall and cotyledon sucrose labeling (fig. S8B); however, the major transported sugar, sucrose, did not correspond to the measured pool sizes of sugars in the endosperm (fig. S9). The endosperm borders the pod wall and cotyledons but was not intimately tied to assimilate delivery, a finding that parallels studies in rapeseed (*39*).

Uniformly labeled malate and glutamine distinguished direct transport from indirect (via pod wall) transport of other metabolites to the cotyledon. Labeled malate resulted in a considerable M4 (fully labeled) isotopologue in the pod wall (Fig. 6C) but not in the cotyledon. When U-^13^C_5_ glutamine was supplied, the isotopologue distribution in malate was also different between these two tissues, indicating that malate was not directly moved from the phloem or pod wall to seed tissue. Conversely, the provision of labeled glutamine resulted in uniform labeling of this amino acid in both tissues, indicating that glutamine was transported intact between pod and cotyledon. The presence of M3 glutamine in both tissues from labeling with U-^13^C_4_ malate indicated that malate was processed through the TCA cycle in the pod wall and used to make glutamine that was further transported to the cotyledon. Labeled alanine provision did not result in label enrichment in any cotyledonary metabolites (data S7).

### Multi-tissue flux analysis describes synergy of pod components and confirms 33 to 45% contribution of pod assimilated carbon to seed filling

A network for isotopically nonstationary metabolic flux analysis (INST-MFA) was constructed on the basis of the eight labeling studies to evaluate and mechanistically quantify the carbon partitioning and synergy between reproductive structures during seed filling (data S7 and S8). The production rate of biomass components was bounded by measured values for the cotyledons as levels in the pods did not change during the seed-filling period (data S6). The modeled metabolic network included two separate locations for sugar metabolism and the CBC in the pod wall as indicated by labeling studies (fig. S10). The exterior surface of the pod wall is primarily responsible for ambient CO_2_ fixation, and the interior surface is heterotrophic but may fix a small amount of seed-respired CO_2_ analogous to reports of nonleaf photosynthesis (*4*, *6*, *40*–*42*). The network complexity was limited to what was necessary to reconcile the multiple labeling experiments. The model included subcellular compartmentation of hexose phosphate pools in the cytosol and plastid of cotyledons for carbohydrate and pentose phosphate metabolism, respectively, to resolve differences in isotopologue patterns between UDPG and hexose phosphates. The interaction between pod wall and cotyledon was characterized by the intertissue transport of metabolites including sucrose and glutamine (from pod wall to cotyledon via endosperm) and respired CO_2_ (from cotyledon to pod wall). The ^13^C-glucose from the phloem was not directly transported to the seed (Fig. 6A); however, the uptake of glucose by camelina seeds at earlier growth stages had been previously considered because of the presence of a glucose pool in the liquid endosperm (*21*). In the current study, a large glucose pool was observed in the endosperm of mid-maturation seeds (fig. S9) that can supply unlabeled cotyledonary glucose (92% M0 at 16 hours; Fig. 6A). Hence, the movement of this sugar to cotyledon from the large endosperm pool, which was likely supplemented from the pod wall, was included in the model network.

**Fig. 7.**
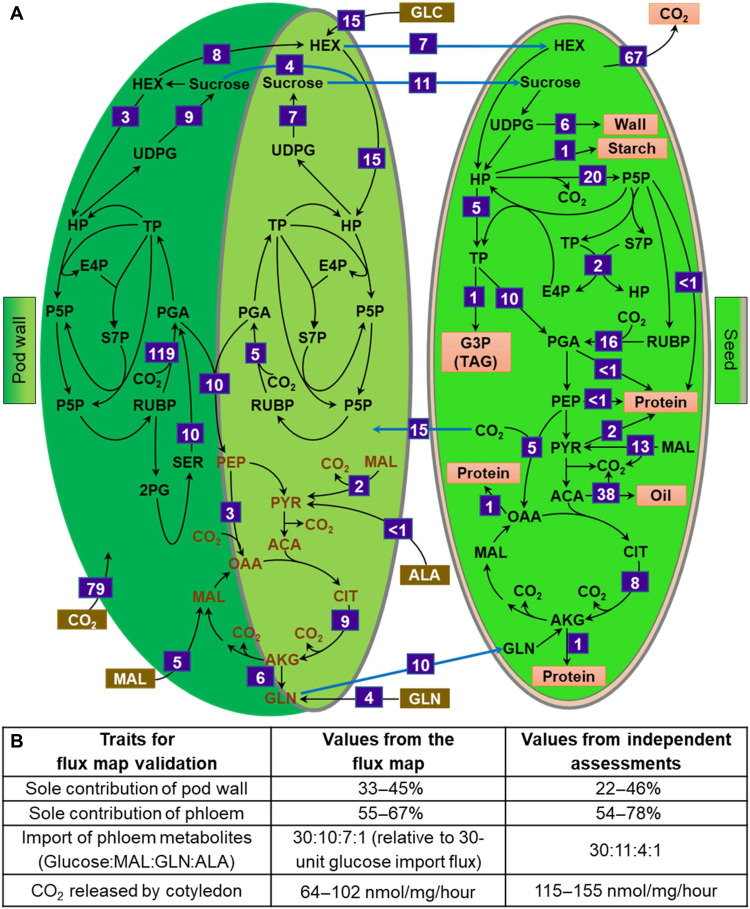
Multi-tissue metabolic flux analysis of a camelina pod system. (**A**) The flux map indicates important carbon fluxes through central metabolism of pod walls and seeds that contribute to seed yield in nmol/mg seed dry weight/hour. Directions of arrows indicate the net flux of a reaction. A subset of fluxes is shown; full details of the network and flux values with intervals at 95% confidence levels are provided in data S9. Aspects of glycolysis and TCA cycle of the pod wall are brown in color, indicating shared pools for exterior and interior pod wall compartments. All abbreviations used are listed in data S3. (**B**) Comparison of synergistic traits derived from flux analysis and independent experimental estimates. The ranges are the lower and upper bounds at the 95% confidence levels for the flux map and means ± SD for the independent assessments.

The camelina multi-tissue flux map corroborated the pronounced contribution of the pod wall photoassimilate (33 to 45%) to seed carbon economy (Fig. 7 and data S9) during the midseed filling period. Pod walls assimilate carbon at approximately 30% of the level of leaves per unit area (Fig. 2D); however, leaf-assimilated carbon contributes to all sinks whereas the pods contribute exclusively to seed filling, resulting in a greater relative contribution (68% of the vegetative contribution). The flux map was validated through independent measurements not used in the modeling effort, including LC-MS/MS–based measurements of the uptake ratio of phloem metabolites and LI-COR–measured CO_2_ release by the cotyledon (Fig. 7B). The map established the imported carbon to the cotyledons to be 19% glucose, 59% sucrose, and 22% glutamine (data S9). Both interior and exterior regions of the pod wall participated in the nutrient supply to cotyledons. The net flux measurements indicated that the metabolism of the interior pod wall surface was heavily influenced by the phloem-imported metabolites while the metabolism of the exterior surface was influenced by ambient CO_2_, although movement of phloem sugars to both pod wall surfaces was unconstrained in the model framework. A limited amount of CBC flux was observed in the interior pod wall that used seed-respired inorganic carbon; however, carbohydrate-related metabolites (e.g., sucrose, UDPG, and hexose phosphates) that were enriched in the inner surface and CBC intermediates (e.g., PGA, S7P, and RUBP) that were enriched in the external surface of the pod wall (data S9) suggested greater heterotrophic metabolism of the interior pod wall and autotrophic metabolism of the exterior pod wall.

### Camelina pods efficiently use carbon for seed development

**Fig. 8.**
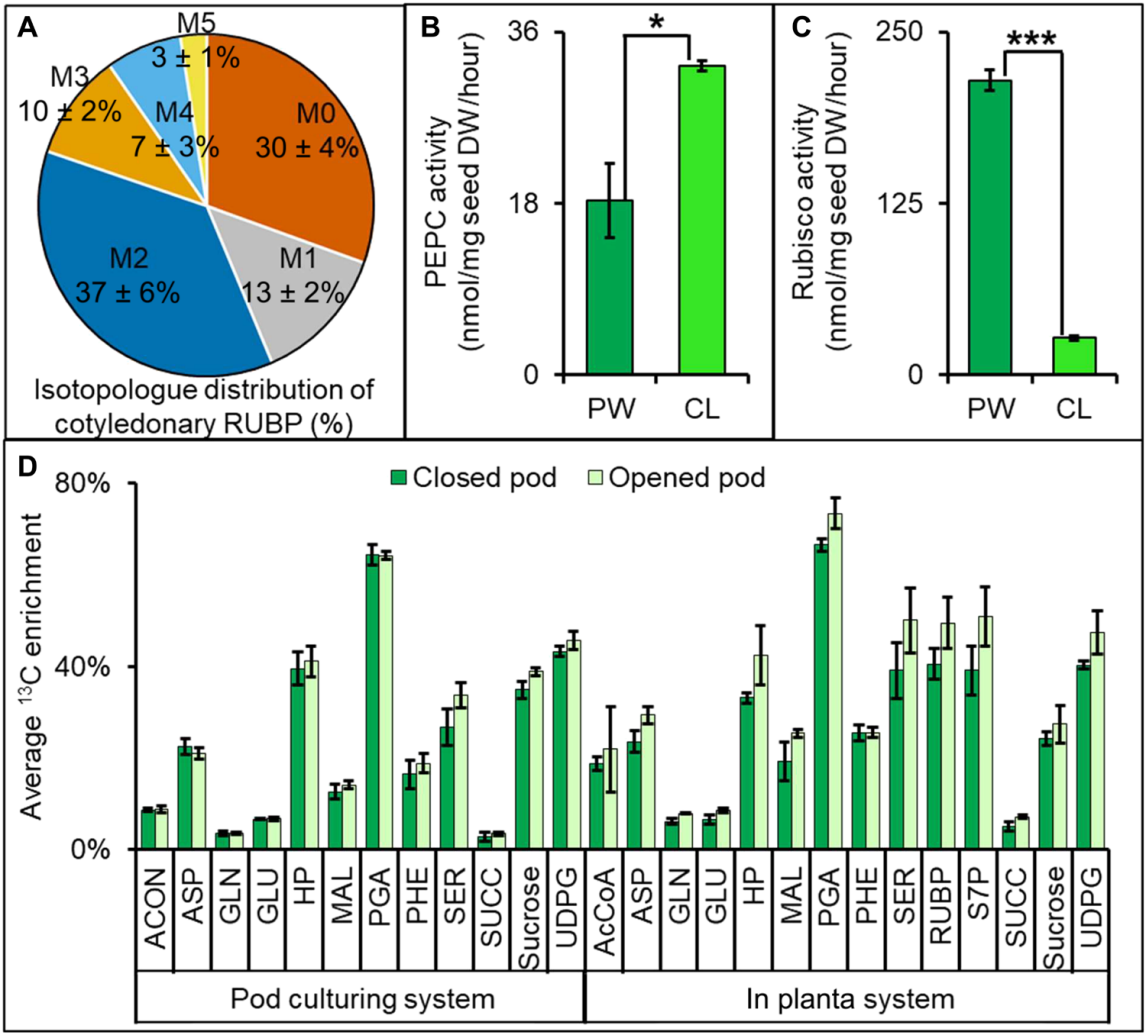
Reassimilation of seed-produced CO_2_. (**A**) Isotopologue distribution of RUBP in the cotyledon after 16 hours of labeling from a supply of U-^13^C glucose. (**B**) PEPC and (**C**) Rubisco enzyme activity assays of the pod wall and seed. Student’s *t* tests were used for statistical analyses of enzyme activity assays between pod walls and seeds; *P < 0.05 and ***P < 0.001. (**D**) Isotopic enrichment in pod wall metabolites from a ^13^CO_2_ source in intact versus opened pods. ^13^CO_2_ gas (350 parts per million) was supplied for in planta studies while ^13^C-bicarbonate released by sulfuric acid was used to generate 1% ^13^CO_2_ inside an Erlenmeyer flask for labeling studies. Comparison of the average ^13^C enrichment in metabolites from intact and opened pods was not statistically different (Student’s *t* test at *P* = 0.05). The results are presented as means ± SE (*n* = 3).

Carbon use efficiency of young camelina seeds was reported to be low [32 to 40%; (*21*, *43*)] relative to other green oilseeds [80 to 93%; (*16*, *17*, *28*, *44*)], with levels comparable to nongreen seeds like sunflower and maize [50 to 65%; (*20*, *29*)]. However, when pod walls are considered, camelina seeds convert 63% of imported carbon to biomass (data S9), reflecting the synergy of tissues. The main CO_2_ releasing biosynthetic pathways in seeds (the oxidative pentose phosphate pathway, oil biosynthesis, and the TCA cycle) resulted in the release of 46.5% of the carbon imported to cotyledons; however, 9.4% (i.e., 9.4 of 46.5 or approximately one-fifth of the respired carbon) was recovered inside this seed tissue. This limited reassimilation in the cotyledon was supported by detection of labeled ribulose 1,5-bisphosphate (RuBP; Fig. 8A), which is a hallmark for CO_2_ reassimilation as RuBP is a substrate for Rubisco activity and not used for other metabolic steps. The reassimilation capacity in the cotyledon was further confirmed by the presence of functional carbon assimilating enzymes Rubisco and phosphoenolpyruvate carboxylase (PEPC; Fig. 8, B and C). PEPC activity was comparable between pod components while Rubisco was elevated significantly in the pod wall, consistent with the described photosynthetic role.

CO_2_ reassimilation by the interior pod wall is thought to be significant in chickpea (*2*) but low in canola (*40*). The camelina flux map indicated that a portion of the seed-released CO_2_ could also be fixed by the pod wall (approximately 7% of the total carbon received by cotyledons; data S9). The results conclusively show for the first time the benefit of pod architecture on the metabolic efficiency of the system, collectively defining an emergent property of plant tissues. Thus, as a system, an intact pod uses 70% of the carbon directed to the cotyledons with the remainder lost to the atmosphere. U-^13^C_4_ malate labeling of intact pods corroborated pod-based reassimilation as pod metabolites including serine, phenylalanine, hexose phosphates, UDPG, and sucrose became singly labeled (M1 isotopologue) from Rubisco-based reassimilation of respired ^13^CO_2_ that was generated from labeled malate by malic enzyme (fig. S4). To better understand the reassimilation efficiency of the interior pod wall, the previously described ^13^CO_2_ provision to intact and opened pods was further analyzed. In the opened pod wall study, the inner pod surface was in direct contact with ^13^CO_2_; therefore, the amount of ^13^C would be higher in metabolites if the inner surface assimilated a substantial amount of CO_2_. However, the difference in labeling between open and intact pod studies was small, regardless of the CO_2_ concentration supplied (Fig. 8D), qualitatively consistent with the limited but non-negligible reassimilation in the inner pod wall quantified in the metabolic flux map (Fig. 7).

The metabolic underpinnings of the nutrient exchange between pod components are explained in detail in this report of camelina pod metabolism. Despite the reduced surface area and stomatal density, the chlorophyll-enriched pod elevated above the canopy can contribute substantially (33 to 45%) to the seed carbon economy. The photosynthetic capacity of this green reproductive tissue compensates for the reduced capacity of senescing leaves because pods are developmentally staged immediately before seed-filling, thus providing a “just in time” temporal delivery of resources that is spatially close to seeds and obviates energetic costs in maintaining gradients to support assimilate translocation from the leaves. It is intriguing to consider the consequences of timing and local delivery of nutrients that likely establish a high number of viable seeds and play a crucial role for seed production in adverse conditions such as drought due to the reduced stomata on pod walls. The coordinated synergistic use of plant tissues that capture and partition carbon effectively within environmental constraints is an emergent property of the plant because it capitalizes on coordinated activity across cell types, tissues, and organs. As leaves and pods can independently support nearly 80% of total seed yield, the combination implies an excess photosynthetic capacity. Leaves are the source of carbon for many developing tissues whereas pod photosynthate is directed to the seed. Thus, engineering sink strength to make better use of the available assimilation capacity could result in greater plant productivity or perhaps serve to sustain growth and resilience in environments when the capacity to capture carbon is more limited.

## MATERIALS AND METHODS

### Plant growth conditions and chemicals

Camelina plants (var. Suneson) were grown at the Donald Danforth Plant Science Center. Greenhouses were maintained at 22°/20°C (day/night), 40 to 50% relative humidity, and a 16-hour day/8-hour night photoperiod including a minimum light level of 250 μmol m^−2^ s^−1^ from a combination of 600-W high-pressure sodium and 400-W metal halide bulbs as supplemental lighting. Natural light varied extensively over the seasons; however, in the summer season, shade cloths helped to maintain a maximum of 500 μmol m^−2^ s^−1^ on bright days. Isotopic tracers were obtained from Sigma-Aldrich (St. Louis, USA) and Cambridge Isotope Laboratories (Tewksbury, USA). Other chemicals and organic solvents were obtained from Thermo Fisher Scientific (Waltham, USA) and Sigma-Aldrich.

### Phenotypic measurements

Net CO_2_ assimilation of camelina leaves and pod walls was measured using the LI-6800 portable photosynthetic system (LI-COR Biosciences, Lincoln, USA). Gas was supplied at a CO_2_ concentration of 400 parts per million at 400 μmol m^−2^ s^−1^ light and 25°C. All seeds were removed to measure the net assimilation in the deseeded pod. CO_2_ respired from camelina seeds was measured at a light level of 20 μmol m^−2^ s^−1^ on a LI-6400 system using an attached insect respiration chamber (catalog no. 6400-89) following the manufacturer’s protocol.

Quantitation of chlorophylls and carotenoids involved powdering leaf and pod wall samples, mixing with 80% acetone, and incubating on a shaker for 1 hour at room temperature followed by overnight dark settling. Sample tubes were covered with aluminum foil to inhibit any light-mediated degradation. After overnight settling, clean supernatant was collected and run on a spectrophotometer (SpectraMax M2; Molecular Devices LLC., San Jose, USA) to measure absorbance at 470, 647, and 663 nm. Acetone (80%) was used as the blank for background measurement. Calculation of phytopigments was performed as previously described (*45*).

The surface area of total leaves and pod walls was calculated using ImageJ software (version 1.53e, Wayne Rasband and contributors, National Institutes of Health, USA). Both sides of the leaf and the outer surface of the pod wall were considered for green surface area measurements. Senescing leaves were excluded from calculations of phytopigments and leaf surface area.

### Pod culturing technique

To mimic in planta conditions in detached pods, phloem sap was collected. Reproductive branches of growing camelina plants were scored with a scalpel and a micropipette used to extract phloem sap. Ten microliters of phloem sap was collected in a tube containing methanol-water (50:50), flash-frozen in liquid nitrogen, and stored at −80°C until further analysis. The entire process was performed within 30 s to minimize enzymatic activities from wound responses or other artifacts.

Culture medium for pods contained modified Linsmaier and Skoog medium (*46*, *47*) along with Gamborg’s vitamins and 5 mM MES buffer. The medium was supplemented with components of phloem sap (glucose, malate, glutamine, and alanine), and the pH was adjusted to 5.8. Images of several labeling approaches are presented in fig. S11. Pods with stems were excised under sterile water and incubated in a 96-well plate where the stem was immersed in the medium. Cultures were nourished in a Percival growth chamber (E22L; Percival Scientific Inc., Perry, USA) with controlled temperature (22°C) and light intensity (usually 250 μmol m^−2^ s^−1^, varying in certain light-dependent studies). ^13^C-bicarbonate studies were performed in 250-μl Erlenmeyer flasks. The amount of supplied ^13^C-bicarbonate (200 μg CO_2_/hour/cm^2^) was varied to match the duration of the experiment and calculated on the basis of the net assimilation of deseeded pods. CO_2_ gas was released inside the flask from bicarbonate powder by the addition of sulfuric acid at the start of the experiment. In planta ^13^CO_2_ labeling studies were performed as previously described (*11*, *48*) using transparent plastic bags as labeling chambers. All isotopic studies are outlined in data S10. For each labeling experiment, the ^13^C tracer was provided as 100% of that metabolite in the medium (e.g., in the case of U-^13^C_6_ glucose labeling, all unlabeled glucose was replaced by labeled glucose while other sources remained unlabeled). Pod culturing with all unlabeled sources was performed for determining pool sizes, biomass compositions of different tissues, and as controls for labeling studies. Samples were flash-frozen in liquid nitrogen after culturing and stored at −80°C. The part of the reproductive branch used for in planta labeling studies was excised immediately before removing the bag and flash-frozen as an intact branch in liquid nitrogen as the bag was being removed. Cultured pods were quickly severed and immediately quenched in liquid nitrogen. In each case, care was taken to avoid shading and minimize the quench period from the point of removal from the labeling apparatus to 1 s or less. Although limited metabolite turnover could occur even within this short duration, it would be less than in leaves for the less responsive photosynthetic pod system. Three sets of tissues from each pod (i.e., pod wall, seed coat, and cotyledon) were dissected on dry ice before extraction.

### Measurement of substrate uptake rate

After 16 hours of continuous culture, the liquid of remaining medium was collected in an Eppendorf tube. Known quantities of ^13^C-based glucose, malate, glutamine, and alanine tracers were added, and the contents were quantified. Spent media was pooled from 12 biological replicates for precise determination. A total of six pooled replicates were collected from six different sets of experiments. Spent media concentration was measured by LC-MS/MS through comparison of the peak area of a labeled standard of known concentration with the unlabeled peak areas of spent media samples, with the spent concentrations subtracted from the known concentrations of precultured medium components to calculate the uptake rate.

### Extraction of hydrophilic metabolites from different pod tissues

Metabolite extraction was carried out as previously described (*49*) using 300 μl of 50 mM aqueous ammonium formate buffer (pH 3), 900 μl of dichloromethane-ethanol (2:1), and internal standards (Pipes, norvaline, and ribitol). The aqueous phase was freeze-dried overnight, resuspended with 50 μl of methanol-water (1:1), and passed through a 0.45-μm centrifugal filter (Costar, Corning Inc.). The filtered solution was then transferred to insert glass vials (Agilent, Xpertek) for LC-MS analysis. When lipid and protein fractions were required, 400 μl of 100% methanol (−20°C) was added to the remaining 600 μl of organic phase (containing lipids and a protein layer), vortexed, and centrifuged at 21,000*g* at 4°C for 10 min, resulting in a protein pellet and a supernatant containing hydrophobic metabolites. The pellet was dried in a CentriVap vacuum concentrator (FreeZone 2.5; Labconco, Kansas City, USA) for protein quantification. The supernatant was dried under nitrogen gas before lipid analysis.

### Measurement of soluble sugars, amino acids, and sugar phosphates

The aqueous phase extract was analyzed by LC-MS/MS using a Shimadzu HPLC system (UFLC-XR; Shimadzu Corporation, Kyoto, Japan) connected to an AB Sciex triple quadrupole MS system (QTRAP 6500; AB Sciex, Foster City, USA). Amino acids and sugars (*50*, *51*) were separated on an Infinity Lab Poroshell 120 Z-HILIC column (2.7 μm, 100 × 2.1 mm; Agilent Technologies, Santa Clara, CA, USA), while sugar phosphates and organic acids (*52*) were separated on an Imtakt Intrada Organic Acid column (150 × 2 mm, 3 μm; Kyoto, Japan). Amino acids were detected in positive ion mode while sugars, sugar phosphates, and organic acids were detected in negative ion mode following previously described methods and MS parameters (*50*–*52*). For quantification of pool sizes and biomass composition, metabolite concentrations and recoveries were calculated on the basis of calibration curves of the individual standard and the internal standard ribitol, respectively.

### Measurement of protein, lipid, and starch content

To compare between the culture and in planta conditions, 12-day-old alternate pods were either cultured for 6 days or left on the plant to grow. After 6 days, total protein content in cotyledons was quantified by elemental C-to-N ratio analysis as previously described (*24*). The composition of carbon and nitrogen in dry biomass was determined at the Duke Environmental Laboratory in Duke University by dry combustion using a CE Instruments NC2100 elemental analyzer (Thermo Quest, Italia). Individual proteinogenic amino acid content was measured by LC-MS/MS after protein hydrolysis as previously reported (*50*).

The amount of total lipid in cotyledons between the culture and in planta conditions was compared by transesterification of fatty acids to fatty acid methyl esters (FAMEs) as previously detailed (*35*). FAMEs were quantified by gas chromatography–flame ionization detection using a DB23 column (30 m, 0.25 mm i.d., 0.25 μm film: J&W Scientific, Folsom, USA). The GC was operated in a split mode (30:1). The temperature gradient of the oven was programmed as isocratic 1 min at 180°C, followed by ramping from 180° to 260°C at a rate of 20°C min^−1^, and held at 260°C for 7 min. Equilibration time was adjusted to 1 min. Peak areas of FAMEs from the sample were compared to C15:0 and C17:0 triacylglycerol internal standards for quantification. Labeling in different individual lipid species, i.e., PC, phosphatidic acid, phosphatidylglycerol, and phosphatidylinositol, was measured by LC-MS/MS using a previously described protocol (*53*).

Starch fractions were extracted from homogenized and dried tissues using the Megazyme starch assay kit (Megazyme International, Ireland), following the reported protocol (*21*). Quantification of starch was based on a standard curve that was generated using starch standards hydrolyzed alongside pod tissues and corrected on the basis of a second curve generated with glucose standards for determining assay recovery.

### Isotopic data interpretation and metabolic flux analysis

Peak areas of individual isotopologues (M0 to Mn) of metabolites were enumerated from mass spectrometric analysis. After correcting the raw data for background noise and natural ^13^C abundance (*54*), the isotopologue distribution [characteristically referred to in flux literature as the mass isotopomer distribution (MID)] and average isotopic enrichment were calculated using the following formula where *C* and *n* denote the corrected isotopologue abundance and number of carbons present in the metabolite, respectively

$${\text{MID}}_{\left(\text{Mn}\right)}=\frac{C_{\left(\text{Mn}\right)}}{\sum_{i=0}^{n}C_{\left(\text{Mi}\right)}}$$ (1)

$$\text{Average}\;13C\;\text{Enrichment}=\frac{\left\{{\text{MID}}_{\left(M0\right)}\times 0\}+\{{\text{MID}}_{\left(M1\right)}\times 1\}+\ldots .+\{{\text{MID}}_{\left(\mathrm{Mn}\right)}\times n\right\}}{n}$$ (2)

The MATLAB-based Isotopomer Network Compartmental Analysis (INCA) package [Version 2.0; (*55*)] was used for MFA. The reaction network for the flux map was designed on the basis of previous MFA reports on Arabidopsis and camelina leaves and seeds (*21*, *56*, *57*) while the current pod wall model network was created by comparing previous leaf models. MIDs of eight complementary ^13^C labeling experiments (data S6) were simultaneously fitted for the integrated flux analysis. The threshold SD value of MIDs for MFA was set to the highest value among three criteria: actual biological SD, common LC-MS/MS–based technical error (0.01 or 1%), and 5% of the average isotopologue value to confront the variation due to eight different biological studies and different time points during isotopically nonstationary labeling studies. Lists of abbreviations, biomass fluxes, and reactions used in the network model are provided in data S3, S6, and S7. To identify a global best-fit estimate, flux evaluations were repeated a minimum of 30 times starting from random initial values; however, including small changes to aspects of the model over its development, thousands of model estimates were determined. The estimated fluxes were accepted when the obtained variance-weighted sum of squared residuals was below the χ^2^ at a 95% confidence level. The accurate nonlinear statistical uncertainty (at 95% confidence level) of all fluxes in the best global-fit flux map was computed by the parameter continuation method (*58*).

### Microscopy analyses

For stomata count, leaf and pod samples were fixed for 24 hours using 4% paraformaldehyde in 1× phosphate-buffered saline buffer, washed 3× in water, and stained for 1 hour in a 20 μM solution of calcofluor white (catalog no. 29067, Biotium, Freemont, USA). Samples were rewashed 3× in water before microscopy analysis and imaged on a Leica SP8-X (Leica, Wetzlar, Germany) with a 20× HD Plan Apochromat objective lens (numerical aperture 0.70) using 405-nm diode laser excitation, 415- to 645-nm emission, and a HyD detector. Three-dimensional (3D) image stacks were acquired with a pinhole of 1 airy unit, 1024 × 1024 pixels, field of view of 581.25 μm^2^, and a 2-μm *z*-interval. Z-stacks were rendered as a 3D maximum intensity projection, imported into the Fiji image processing platform, and each stoma was marked and counted with the Cell Counter plugin.

Horizontally growing leaves and pods were selected to hold a drop of 1% aqueous Lucifer yellow dye for 10 hours. About 5 mm^2^ of exterior pod or adaxial leaf surface was abraded with fine sandpaper without disturbing the septum of the pod or the large vein of the leaf. Ten microliters of 1% dye (or no dye on the abraded surface for the control pod wall sample) was applied on the abraded surface. Images were acquired on a Zeiss AxioZoom V.16 system using a PlanNeoFluar Z 1× objective lens (0.25 numerical aperture). All corresponding treatment and control images were acquired with a Zeiss Axiocam 512 color camera at 14-bit with 4248 × 2832-pixel images under identical zoom and light intensity and camera settings using 450- to 490-nm excitation and 500- to 550-nm emission filters.

### Enzyme activity assays

The in vitro PEPC enzyme activity was measured using an assay kit following the manufacturer’s protocol (catalog no. ab239720, Abcam, Waltham, USA), and the total protein content for assayed samples was quantified using a Bradford assay kit (catalog no. B6916, Millipore Sigma, Burlington, USA). For the in vitro Rubisco activity assay, powdered samples were extracted by bead beating in 1 ml of 50 mM Hepes (pH 7.8), 1% polyvinylpolypyrrolidone, 1 mM EDTA, 10 mM dithiothreitol, 0.1% (v/v) Triton X, and 2% (v/v) protease inhibitor cocktail (catalog no. P9599, Sigma-Aldrich). Crude extracts were centrifuged at 4°C for 5 min, and the supernatant was collected for the next step. Rubisco activity was spectrophotometrically measured from the conversion of NADH [reduced form of nicotinamide adenine dinucleotide (NAD^+^)] to NAD^+^ by observing the change in absorbance at 340 nm following protocols described previously (*59*, *60*).

### Statistical analyses

Two-tailed unequal variance Student’s *t* tests were performed to understand the significance in the difference in phenotypic traits between leaves and pod walls (Fig. 2, A to C), biomass comparisons between in planta and culture conditions (Fig. 3D), seed yield between control and shaded leaves or pods (Fig. 5B), pool sizes between pod wall and cotyledon (Fig. 6B), enzyme activity assays between pod walls and seeds (Fig. 8, B and C), and average ^13^C enrichment between opened and closed pod conditions (Fig. 8D and fig. S5).

## Notes

View/request a protocol for this paper from *Bio-protocol*.
