# Supplementary material for: Metabolic synergy in Camelina reproductive tissues for seed development
Source: Sci Adv. 2022 Oct 28;8(43):eabo7683. doi: 10.1126/sciadv.abo7683 (PMC9616503; doi:10.1126/sciadv.abo7683)
Supplement: Supplementary file 1 — Figs. S1 to S11 [file sciadv.abo7683_sm.pdf]

Science Advances
AAAS

Supplementary Materials for

Metabolic synergy in Camelina reproductive tissues for seed development

Somnath Koley *et al.*

Corresponding author: Doug K. Allen, doug.allen@ars.usda.gov

Sci. Adv. **8**, eabo7683 (2022)
DOI: 10.1126/sciadv.abo7683

The PDF file includes:

Figs. S1 to S11
Legends for data S1 to S10

Other Supplementary Material for this manuscript includes the following:

Data S1 to S10

Supplementary Figures:

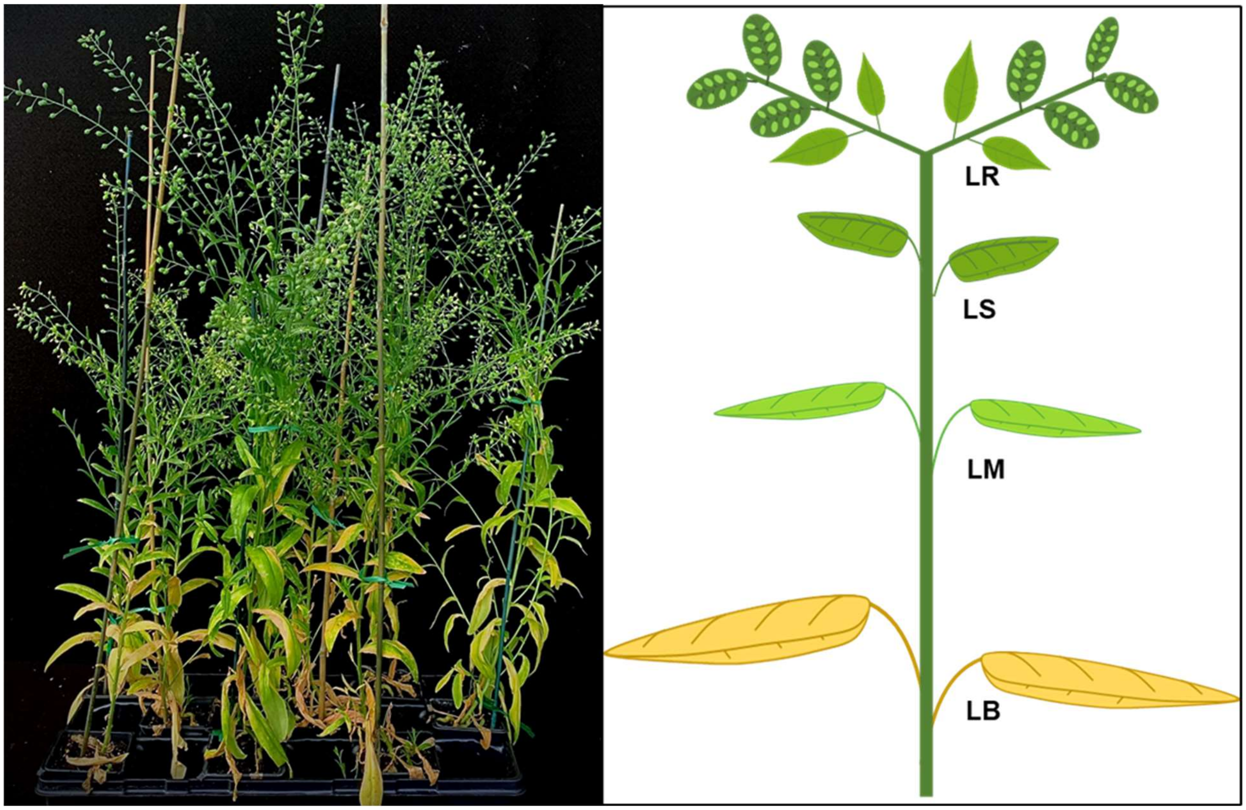

Fig. S1. *Camelina sativa* plants around 15 DAF growth stage. This growth stage occurs 55-65 days after sowing. On the right side, an illustration highlights the position and greenness of leaves (big leaves (LB), medium leaves (LM), short leaves (LS), and leaves at reproductive branch (LR)) and pods.

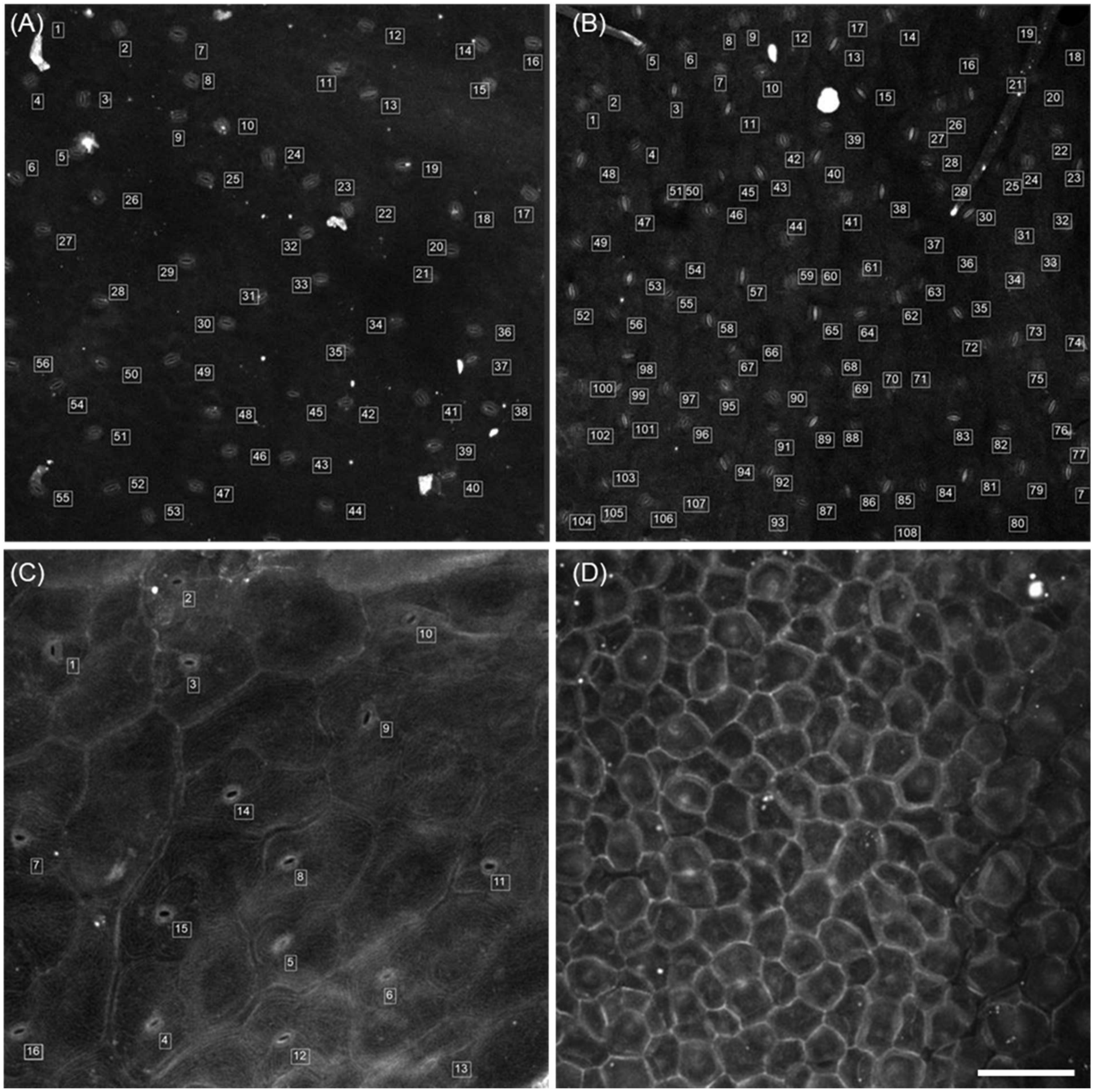

Fig. S2. Images representing stomatal count on the **(A)** abaxial leaf surface, **(B)** adaxial leaf surface, **(C)** exterior pod wall surface, and **(D**) seed surface. Bars = 100 µm.

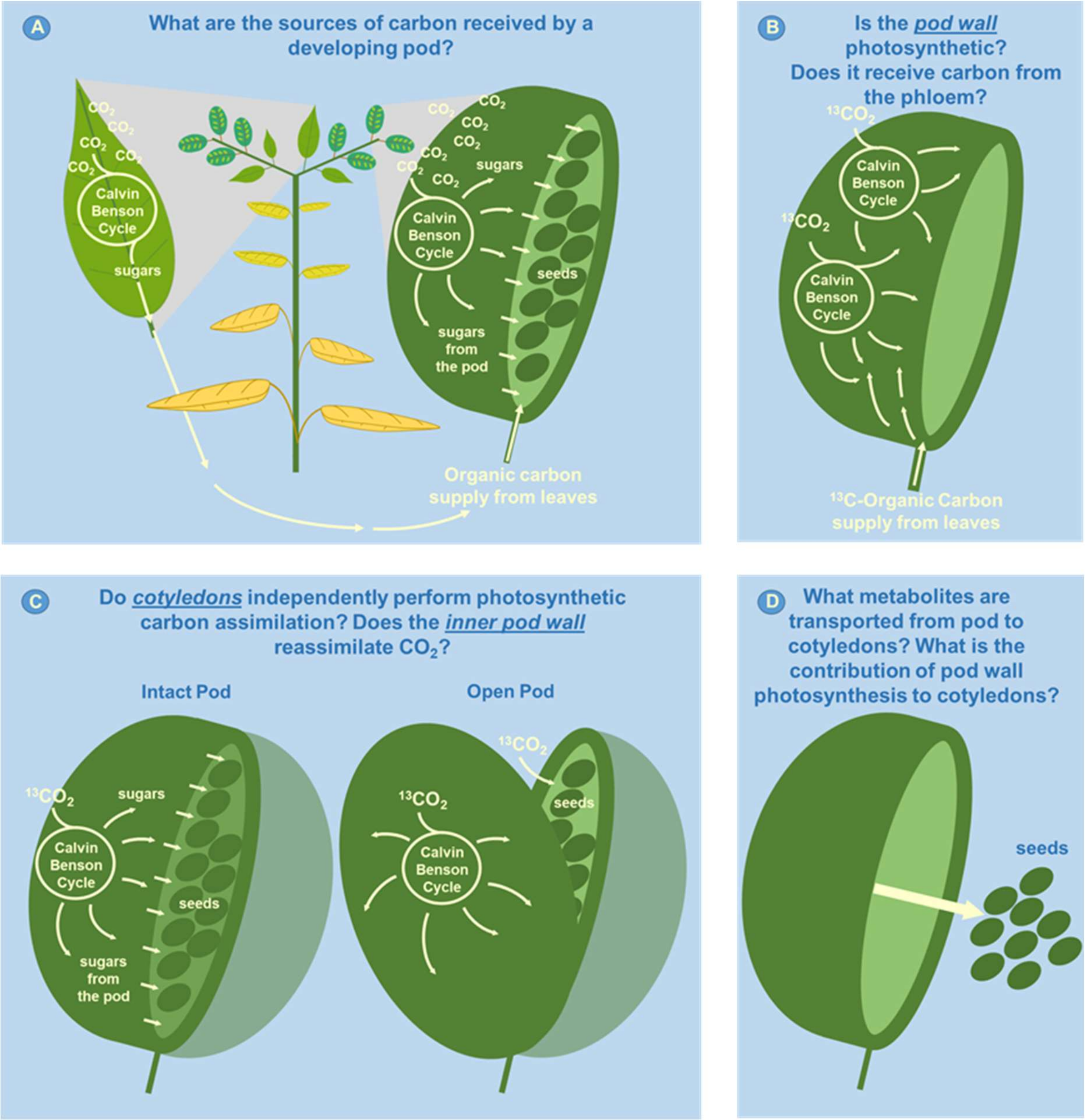

Fig. S3. Logic of isotopic labeling experiments. The purpose of labeling experiments is to 'trace' the incorporation and interconversion of the isotope into different metabolites. Importantly, if natural abundance is accounted for, then the presence or absence of an isotope is an absolute signature for connecting the provided labeled substrate with labeled products, though possibly involving more than one biochemical route through a metabolic network. Deciphering the flux through pathways frequently involves

measurement of pathway intermediates and can be enabled by multiple labeling experiments that introduce an isotope at different locations within the network and therefore report more sensitively on different fluxes in the network. Labeling experiments were designed to assess the contribution and metabolic role of tissues comprising the reproductive pod system in support of metabolic flux analysis. **(A)** Contents of the phloem were examined to determine carbon sources received by a pod. **(B)** $^{13}CO_2$ was supplied to the airspace to evaluate the contribution of photosynthetic carbon assimilation by the pod wall, while ^{13}C-sugars were supplied through the stem to evaluate the phloem contribution. The ^{13}C was tracked into central carbon intermediates in the pod wall tissue. **(C)** The possibility of direct photosynthetic assimilation of carbon by developing seeds and the reassimilation capacity of the inner pod wall was inspected through labeling comparisons of central carbon intermediates in seeds and pod walls, respectively, between open and closed pod experiments **(D)** The carbon sources transported to the cotyledons originating from the phloem or from pod photosynthesis were evaluated by comparing the labeling of metabolites between the pod wall and the cotyledons. Evaluating the ^{13}C in cotyledons from multiple labeling experiments enabled an estimated range for the contribution of pod photosynthesis to total seed carbon. The quantitative assessment of labeling experiments and synergy of tissues was further evaluated through a computational metabolic flux model.

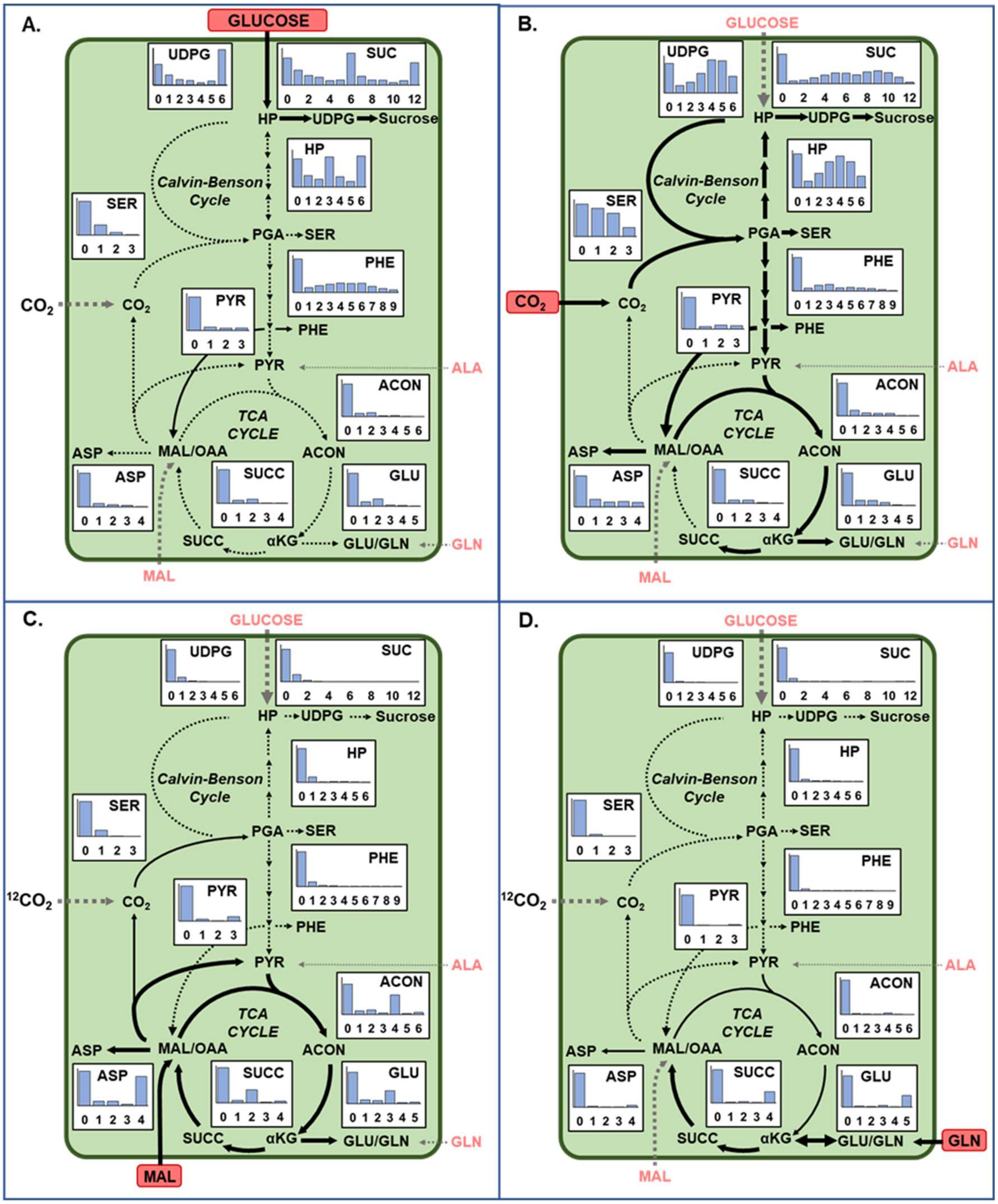

Fig. S4. Detailed labeling phenotypes in different pod wall metabolites at 16 h from four different isotopic sources: **(A)** U-^{13}C glucose, **(B)** $^{13}CO_2$, **(C)** U-^{13}C malate (MAL), and **(D)** U-^{13}C glutamine (GLN). The y-axis represents relative isotopologue distributions and the x-axis represents different isotopologues (M0 to Mn). All results are presented as mean (n= 3). Standard deviations of means are not shown to improve visualization but are provided in Data S7.

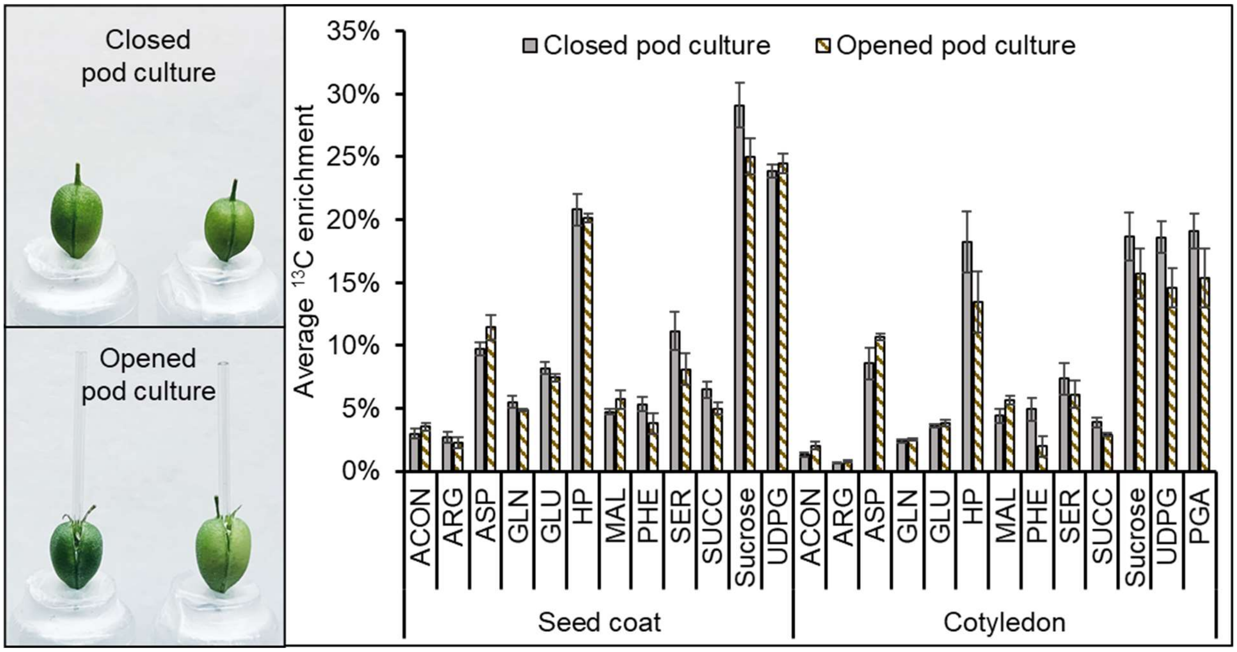

Fig. S5. Isotopic enrichment at inner pod tissues (*i.e.*, seed coat and cotyledon) in intact and opened pod cultures after 8 h of culturing using $^{13}CO_2$ as the labeled source. Results are presented as mean ± SE (n= 3). The average ^{13}C values for all measured metabolites are statistically insignificant between intact and opened conditions (student's t-test at p=0.05).

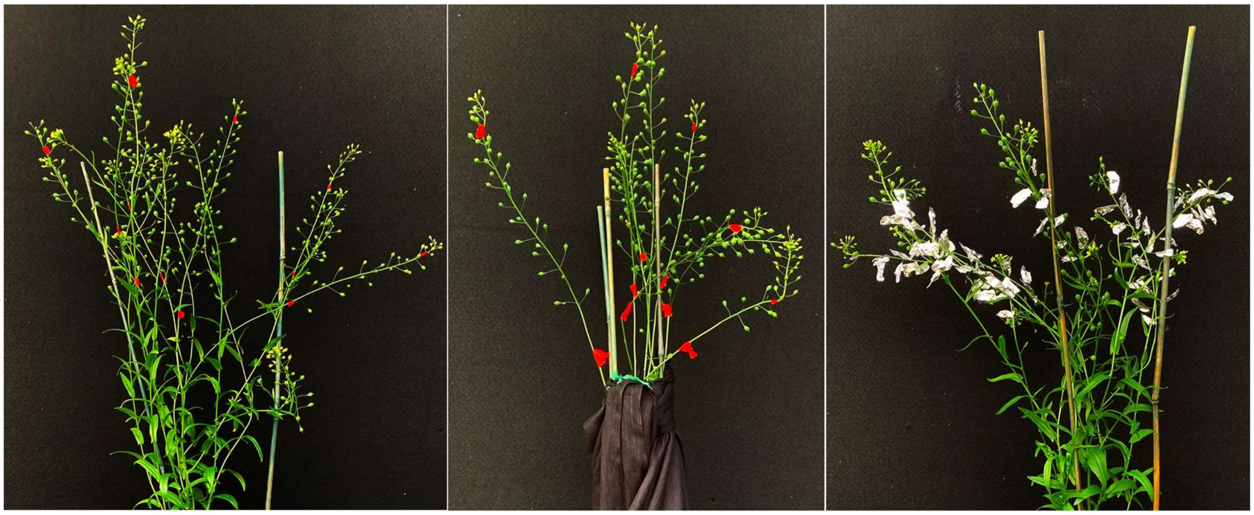

Fig. S6. Shading of all leaves or pod walls *in planta* at the early seed developmental stage. In control plants (left), no leaves or pods were shaded, while leaves using black cloth (middle) and pods using aluminum foil (right) were covered in shaded plants. After 45 days of shading, the biomass of 100 mature seeds was measured using the seeds from pods present at the beginning of the experiment.

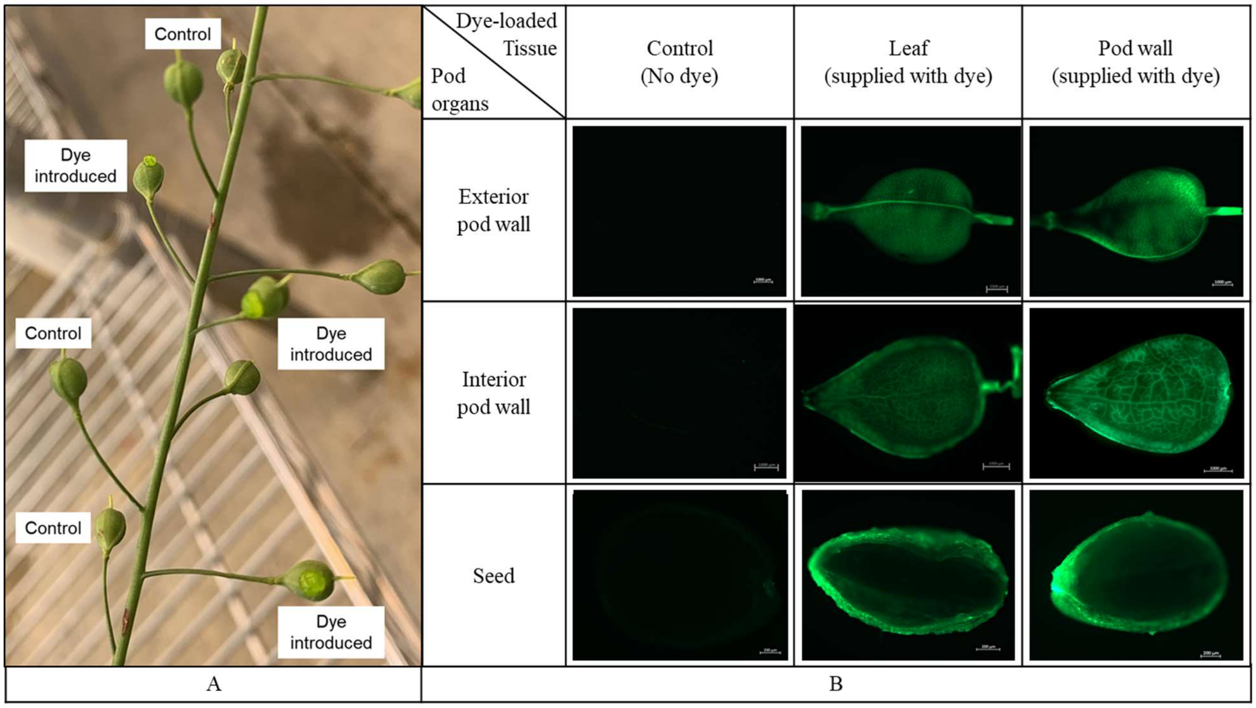

Fig. S7. Application of lucifer yellow fluorescent dye. (A) Control samples where no dye was applied on the abraded pod wall and dye introduced samples where fluorescent dye was applied on the abraded pod wall. **(B)** Detection of fluorescent dye in pod walls and seeds using confocal microscopy after 10 h of lucifer yellow loading onto the upper surface of the leaf or exterior surface of the pod wall. No dye was introduced in the control pod wall sample. Pod wall scale bars = 1000 μm and seed scale bars = 200 μm.

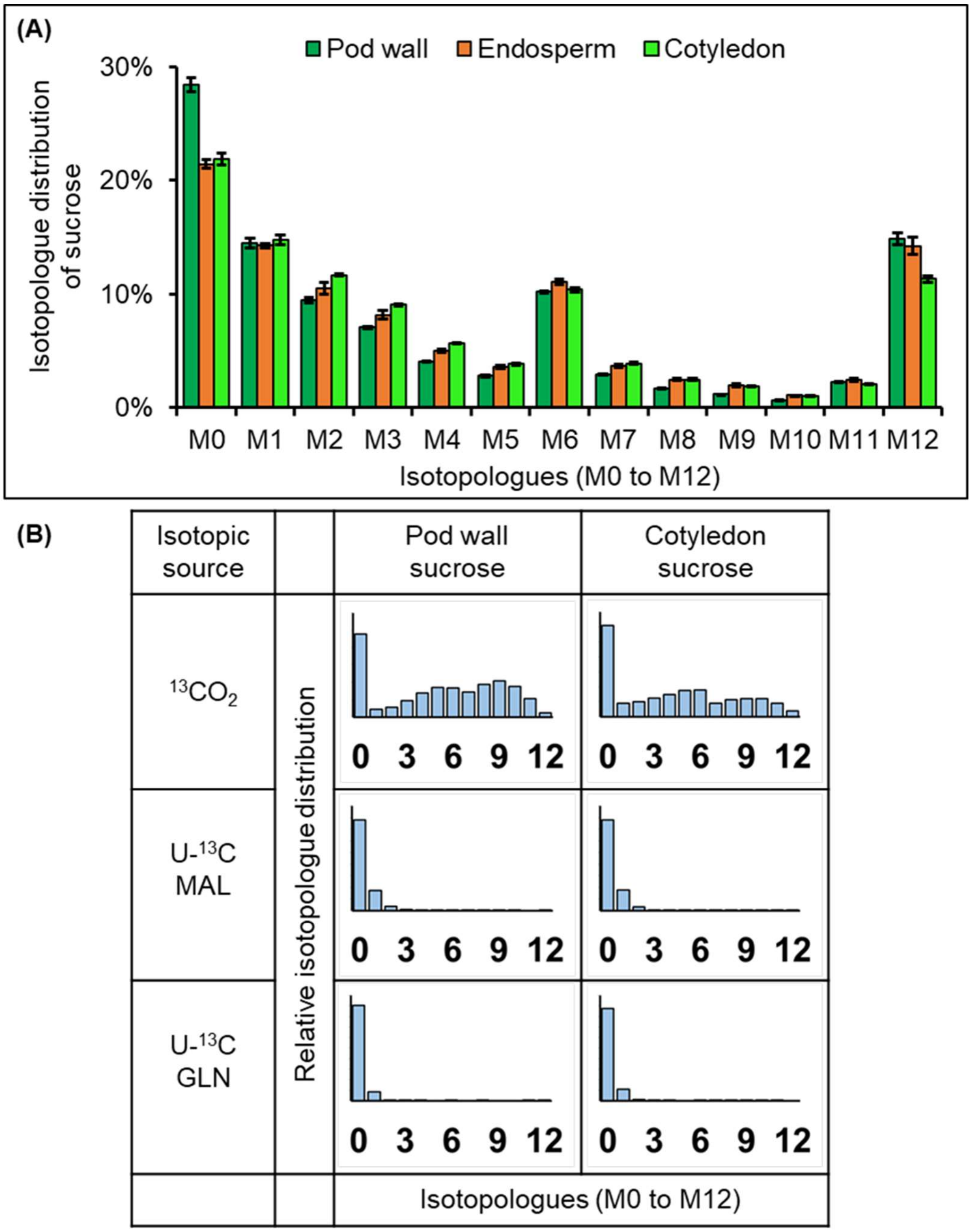

Fig. S8. Isotopologue distribution of sucrose after 16 h of continuous feeding of isotopic source. All results are presented as mean ± SE (n= 3). **(A)** Comparable sucrose labeling patterns among pod wall, endosperm, and cotyledon from a U-^{13}C sucrose source that was supplied in the media instead of glucose at a similar molar carbon amount. **(B)** Comparable sucrose labeling patterns between pod wall and cotyledon from $^{13}CO_2$, ^{13}C-MAL, or ^{13}C-GLN sources. Standard deviations of means are not shown to improve visualization but are provided in Data S7.

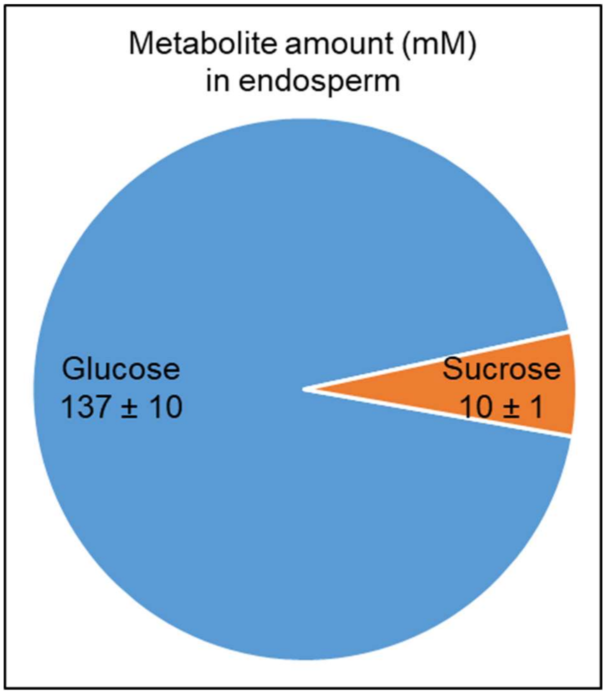

Fig. S9. Concentrations of glucose and sucrose in the liquid endosperm of camelina at 15 DAF growth stage. Results are presented as mean ± SE (n= 3).

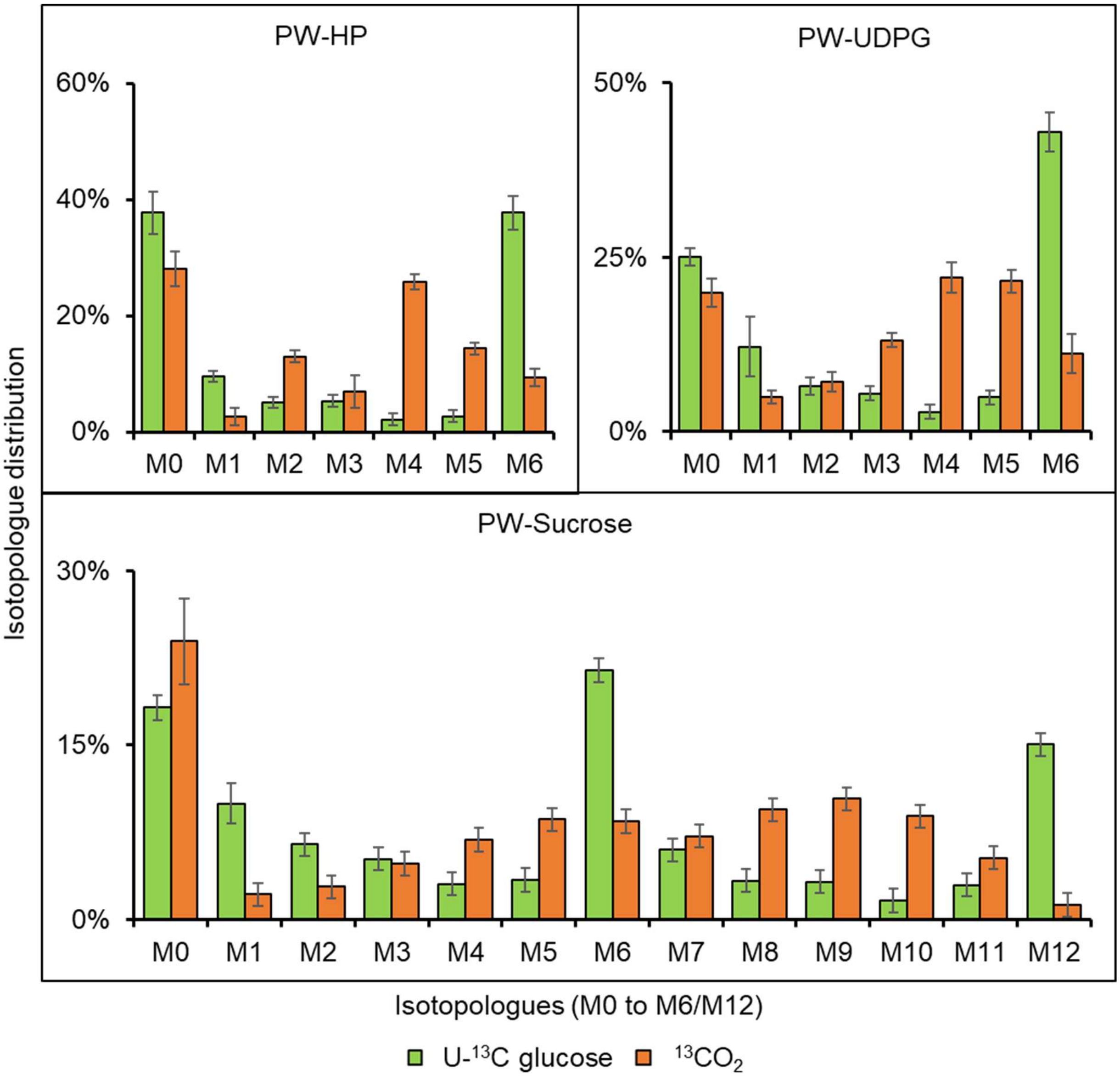

Fig. S10. Isotopologue distributions of pod wall HP, UDPG, and sucrose after 16 h of continuous feeding of U-^{13}C glucose or $^{13}CO_2$. Hexose phosphates are easy to transport between subcellular locations. However, a large untouched pool (M0) from both independent ^{13}C studies signifies metabolism at two separate locations in the pod wall. Similar observations were found in UDPG and sucrose which are produced from HP. Additionally, the labeling patterns of M1 to M5 in HP and UDPG in the U-^{13}C glucose study demonstrated that some isotopic CO_2 is produced from inside the pod wall which was reassimilated by Rubisco in a separate location (*i.e.*,

the M6-operating location). From the $^{13}CO_2$ based study, the pattern was reversed as M6 was not the highest isotopologue even when M3-PGA was the highest (Data S7). This labeling pattern was due to the reassimilation of $^{12}CO_2$ produced from biosynthetic pathways using the unlabeled glucose source. These trends were similar in sucrose, confirming that significant metabolism is occurring in two separate locations of the pod wall.

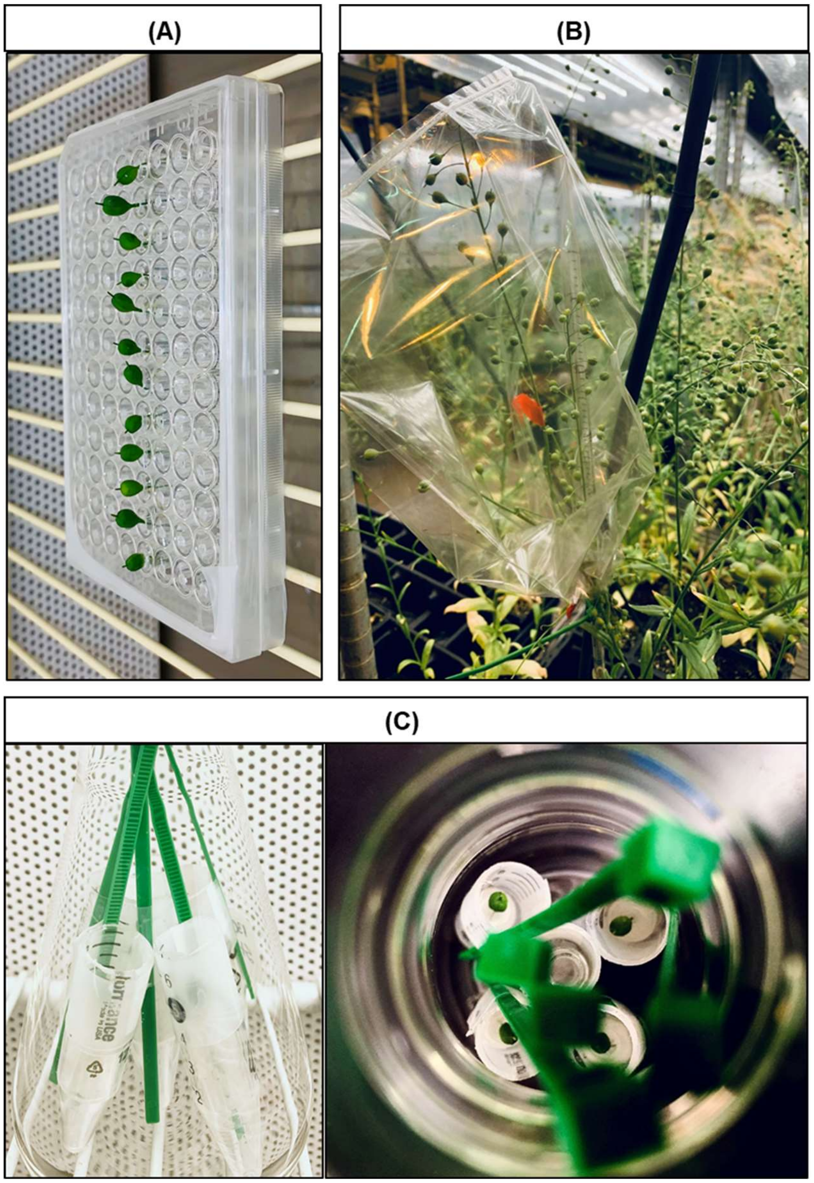

Fig. S11. Images of different pod labeling studies. (A) Pods were incubated in a 96-well plate for studies with unlabeled carbon (control) and ^{13}C-phloem components. **(B)** For *in planta* labeling studies, $^{13}CO_2$ was supplied in a closed transparent bag near the top with a long pipet where all the leaves were removed from the reproductive branch and the bag was closed around the stem at the base where gas was also allowed to exit the system. **(C)** Front (left) and top (right) views of ^{13}C-bicarbonate studies in a 250 mL Erlenmeyer flask where four pods were incubated in four vials and $^{13}CO_2$ was infused in the flask from ^{13}C-bicarbonate by sulfuric acid from the fifth vial located at the middle position.

Captions for Data S1 to S10 (the Microsoft Excel file containing Data S1 to S10 is in a separate supplementary material document)

Data S1. Phenotypic measurements of camelina leaves and pod walls

Data S2. Phloem sap content (molar %) in reproductive branches

Data S3. Abbreviations used in the manuscript

Data S4. $^{13}CO_2$ incorporation in pod wall metabolites in dark vs normal light conditions at 4 h

Data S5. Amount of respired CO_2 from seeds

Data S6. Production rates of seed and pod wall biomass

Data S7. Natural abundance-corrected isotopologue abundances of metabolites used in the ^{13}C INST-MFA models and average ^{13}C percentages of metabolites

Data S8. Complete list of reactions and atom transitions for the multi-tissue camelina pod metabolic network

Data S9. Net and exchange fluxes determined by ^{13}C parallel INST-MFA and flux ratio calculation for the multi-tissue camelina pod metabolic network

Data S10. Objectives of different isotopic studies
